# Gap‐Free Information Transfer in 4D‐STEM via Fusion of Complementary Scattering Channels

**DOI:** 10.1002/advs.76620

**Published:** 2026-07-23

**Authors:** Shengbo You, Georgios Varnavides, Sagar Khavnekar, Nikita Palatkin, Sihan Shao, Mingjian Wu, Daniel Stroppa, Darya Chernikova, Baixu Zhu, Ricardo Egoavil, Stefano Vespucci, Dileep Krishnan, Xingchen Ye, Florian K. M. Schur, Erdmann Spiecker, Philipp Pelz

**Affiliations:** ^1^ Institute of Micro‐ and Nanostructure Research (IMN) & Center for Nanoanalysis and Electron Microscopy (CENEM) Friedrich Alexander‐Universität Erlangen‐Nürnberg, IZNF Erlangen Germany; ^2^ Department of Imaging Physics Delft University of Technology Delft The Netherlands; ^3^ Materials and Structural Analysis Division, Thermo Fisher Scientific Eindhoven The Netherlands; ^4^ DSTL GmbH Baden Switzerland; ^5^ Institute of Science and Technology Austria (ISTA) Klosterneuburg Austria; ^6^ Department of Chemistry Indiana University Bloomington Indiana USA

**Keywords:** 4D‐STEM, dark‐field STEM, data fusion, electron microscopy, ptychography

## Abstract

Linear phase‐contrast scanning transmission electron microscopy (STEM) techniques compatible with high‐throughput 4D‐STEM acquisition are widely used to enhance phase contrast in weakly scattering and beam‐sensitive materials. In these modalities, contrast transfer is often suppressed at low spatial frequencies, resulting in a characteristic contrast gap that limits contrast. Approaches that retain low‐frequency phase contrast exist but typically require substantially increased experimental complexity, restricting routine use. Dark‐field STEM imaging captures this missing low‐frequency information through electrons scattered outside the bright‐field disk, but discards a large fraction of the scattered signal and is therefore dose‐inefficient. Fused Full‐field STEM (FF‐STEM) is introduced as a 4D‐STEM imaging modality that overcomes these limitations by combining ptychographic phase reconstruction with tilt‐corrected dark‐field imaging within a single acquisition. Bright‐field data are used to estimate probe aberrations and reconstruct a high‐resolution phase image, while dark‐field data provide complementary low‐frequency contrast. The two channels are fused in Fourier space using Wiener‐band weighting based on the spectral signal‐to‐noise ratio, yielding transfer‐gap‐free images with high contrast. FF‐STEM preserves the upsampling and depth‐sectioning capabilities of ptychography, adds robust low‐frequency contrast characteristic of dark‐field imaging, and enables dose‐efficient, near–real‐time reconstruction.

## Graphical abstract

Fused Full‐Field STEM (FF‐STEM) is introduced as a 4D‐STEM imaging modality that combines direct ptychography with tilt‐corrected dark‐field reconstruction in a single acquisition. Fourier‐space fusion using Wiener‐type spectral weighting closes the low‐frequency contrast gap inherent to bright‐field methods, delivering gap‐free, dose‐efficient, near‐real‐time images that preserve high‐resolution phase, depth sectioning, and full use of every detected electron.

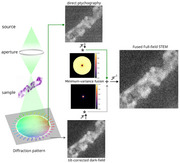

## 1 Introduction

Transmission electron microscopy (TEM) is a central technique for nanoscale materials characterization, providing imaging with atomic‐level resolution. In scanning transmission electron microscopy (STEM), advances in direct electron detector technology [1, 2] now enable the acquisition of a two‐dimensional convergent beam electron diffraction pattern at every probe position, giving rise to four‐dimensional STEM (4D‐STEM) [3, 4]. Due to the rich information encoded in the 4D‐STEM dataset, a variety of reconstruction strategies have been developed to extract structural and phase information of the specimen. Among these methods, iterative ptychography [5, 6] delivers the highest quantitative accuracy and resolution, as it models multiple scattering and probe‐sample interactions through successive forward and backward propagations of the electron wave. Combining with multislice [7] or tomography [8, 9], these iterative schemes have demonstrated sub‐Ångström phase retrieval on bulk‐like materials and even three‐dimensional reconstructions of complex specimens with sub‐Ångström 3D resolution [10].

Simple, real‐time capable approaches such as the center‐of‐mass (COM) [11, 12, 13], differential phase contrast (DPC) [14] or optimum bright‐field (OBF) STEM [15] methods retrieve the projected electrostatic potential using linear approximations to the phase‐retrieval problem.

More advanced techniques enable analytical phase retrieval of the specimen transmission function from the recorded interference between overlapping diffraction disks under the weak‐phase approximation. Direct ptychography methods correct aberrations with a Fourier filter operation before aggregating all information scattered into the bright field regions into a 2D image [15, 16, 17, 18, 19].

Tilt‐corrected bright‐field [20, 21, 22] or parallax imaging [23], enhance phase‐contrast transfer at spatial frequencies smaller than the numerical aperture by compensating for defocus‐induced image shifts, while additional aberrations lead to significant damping of the transferred contrast [24]. These linear bright‐field reconstruction methods inherently suppress low spatial frequency components of the image, leading to reduced contrast in slowly varying specimen regions.

Electrons scattered to high angles and detected in the dark‐field region of the detector, on the other hand, are known to transfer low spatial frequencies well, as they mainly contain incoherent signals [25, 26] when a large detector is used. Tilt‐corrected dark‐field (tcDF) imaging was recently introduced [19] to fully utilize the detected electrons, also with linear imaging methods. By grouping electrons scattered to high angles into segmented dark‐field regions and compensating for defocus‐induced image shifts, tcDF reconstructs images that emphasize slowly varying structural features and mass–thickness contrast. This approach is robust for thick specimens or regions affected by dynamical scattering, where bright‐field‐based methods often fail to accurately recover the projected potential due to the breakdown of the single‐scattering (weak phase object) approximation under dynamical diffraction conditions. Previous work by Yang et al. [27] demonstrated simultaneous ptychographic and high‐angle annular dark‐field (ADF) imaging from 4D‐STEM data, though without fusing the two channels into a single reconstruction. However, while tcDF enhances low‐frequency contrast, it does not recover phase information accessible through ptychography. Therefore, combining the complementary strengths of direct ptychography and tilt‐corrected dark‐field imaging provides a promising way toward a unified reconstruction framework that preserves both high‐ and low‐frequency contrast in 4D‐STEM. In this work, “direct ptychography” refers specifically to the aberration‐corrected Fourier‐domain reconstruction based on the normalized aperture‐overlap function $\Gamma^{*}/\left|\Gamma \right|$ as detailed in the Methods.

Building upon the complementary contrast mechanisms of bright‐field phase‐contrast methods and dark‐field imaging, we introduce the Fused Full‐field STEM (FF‐STEM) reconstruction method which integrates information from both direct ptychography and tcDF imaging, while preserving the capability of upsampling, depth sectioning and near‐real‐time reconstruction. In this approach, the bright‐field channel contributes phase information and high‐frequency detail, while the dark‐field channel restores the low‐frequency signal missing from direct ptychography reconstructions. The fusion is performed in Fourier space, guided by Wiener‐type weighting functions derived from the spectral signal‐to‐noise ratio (SSNR) of both signal channels, ensuring a smooth and physically consistent transition between spatial frequency regimes. In addition, our implementation incorporates aberration determination through analytical gradient backpropagation using an image quality metric. For megapixel images, all operations are executed in less than half a second on modern parallel processing hardware. As a result, FF‐STEM achieves high‐contrast imaging that unites the strengths of both reconstruction modes while retaining near‐real‐time performance.

## 2 Results

### 2.1 Full‐Field STEM Recovers High‐Contrast and Fully Dose‐Efficient STEM Images With Gap‐Free Information Transfer

**FIGURE 1:**
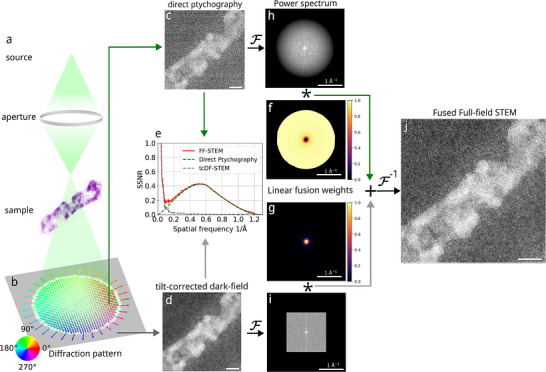
Schematic illustration of the FF‐STEM reconstruction workflow (a) Simplified 4D‐STEM acquisition scheme in which a convergent electron probe raster scans through the specimen while a two‐dimensional diffraction pattern is recorded at each scan position, the sample is a ${\mathrm{Gd}}_{2}O_{3}$ nanohelix. (b) Example diffraction pattern showing the bright‐field (white) and dark‐field (gray) regions. The arrows represent the calculated shifts. (c) Aberration‐compensated direct ptychography image (d) tcDF image reconstructed by correcting image shifts of the dark field segments indicated by color‐coded arrows in (b). Next, the spectral signal‐to‐noise ratio of both channels is calculated (e) and Wiener‐type minimum variance spectral weight for each channel is derived from both SSNRs. (f) and (g) show the spectral weights of ptychography and tcDF, respectively. The Fourier spectra of both channels (h,i) are filtered with the weights from SSNR and are then summed in the Fourier domain and inverse‐transformed to yield the final (j) FF‐STEM image that utilizes all detected electrons. Scale bars in real space images are 5nm.

Figure 1 illustrates the principle of FF‐STEM. A convergent probe interacting with a nano‐spiral specimen produces a 2D diffraction pattern containing a bright‐field (BF) disk and surrounding dark‐field (DF) region (Figure 1a). Raster scanning the probe yields a 4D‐STEM dataset containing both spatially resolved diffraction and real‐space contrast (Figure 1b).

The BF disk contains the coherent interference between the unscattered probe and beams diffracted within the aperture, as described in the first‐order (single‐scattering) approximation [28]; it therefore carries the coherent amplitude and phase information used by direct ptychography and other BF‐based STEM phase‐retrieval methods. However, this channel suffers from a well‐known dip at low spatial frequencies because only interference of real‐space points lying inside the small probe illumination window is encoded linearly in the diffraction signal [29]. In contrast, the DF region contains strong low‐frequency information arising from incoherent amplitude scattering in the dark‐field angular range [19, 28]. Simultaneously, azimuthal dark field segments also display image shifts depending on the defocus aberration of the probe. These shifts encode low‐frequency and depth information but are usually lost when the DF signal is summed incoherently [19].

FF‐STEM unifies these two complementary sources of information. Any aberrations present in the BF region are algorithmically determined and corrected using the aberration gradient obtained from an image‐contrast metric of the ptychography image (Methods, Equation (5)), while the DF region is corrected using tilt‐corrected dark‐field (tcDF) imaging (Methods, Equation (18)), which removes the defocus‐induced real‐space shifts associated with each azimuthal DF segment [19]. After these corrections, the BF‐based ptychographic image and the DF‐based tcDF image carry complementary contrast functionals of the same specimen, sampled in disjoint spatial‐frequency bands: the ptychographic channel is dominated by the coherent phase term proportional to σV, while the tcDF channel encodes the incoherent amplitude term proportional to $\sigma^{2}V^{2}$ [19].

To combine these reconstructions, we estimate the spectral signal‐to‐noise ratio (SSNR) of each modality. For weak‐phase direct ptychography, the SSNR follows a known analytical form that depends only on the probe aperture and aberration surface [29, 30] (Methods, Equation (13)). For tcDF, the signal is object‐dependent and no analytical SSNR exists [19, 28]; we therefore estimate it from two independent half‐data tcDF reconstructions using the established half‐split approach from cryo‐EM [31, 32] (Methods, Equation (24)). This provides unbiased estimates of the signal and noise power spectra of the tcDF channel.

The two imaging modalities are then fused in Fourier space using Wiener‐type weights derived directly from their SSNRs (Methods, Equation (39)). At each spatial frequency, the weight automatically selects the channel with the higher spectral reliability: tcDF dominates at low spatial frequencies, where ptychography is intrinsically suppressed, while ptychography dominates at higher frequencies, where tcDF carries little coherent signal. The fusion therefore yields continuous, gap‐free contrast transfer across the entire spatial‐frequency range.

Let $\hat{O}\left(q\right)$ and ${\hat{O}}_{\mathrm{tcDF}}\left(q\right)$ denote the Fourier transforms of the ptychography and tcDF reconstructions. The FF‐STEM image in Figure 1j) is obtained by inverse Fourier‐transforming the weighted sum of these spectra (Methods, Equation (40)). Because the noise contributions of the two channels are statistically independent, the noise variance of the FF‐STEM image combines inverse‐additively across channels (Methods); the per‐channel SSNRs and their sum are shown in Figure 1e as a summary diagnostic of the complementary frequency‐domain information content of the two channels.

Applied to the nanohelix dataset in Figure 1, FF‐STEM yields a high‐contrast image with signal transfer from the lowest spatial frequencies to the edges of twice the numerical aperture limit. The resulting SSNR curve (Figure 1e) shows complete removal of the low‐frequency dip characteristic of ptychography and other BF‐STEM imaging methods, confirming that FF‐STEM restores information that is so far not recovered by bright‐field STEM imaging methods.

### 2.2 Contrast Transfer and Depth Sectioning Using FF‐STEM

#### 2.2.1 Recovery of Low‐ and High‐Frequency Information in Simulation

To evaluate the performance of the FF‐STEM method, we simulated a ${\mathrm{Co}}_{3}O_{4}$ nanoparticle with a fluence of $1.0\times 10^{3}e/{Å}^{2}$. The aberrations of the 4D‐STEM dataset are calculated using our gradient backpropagation approach (Methods, Equation (8)). With the calculated aberrations, the direct ptychography reconstruction and tcDF are performed independently. For the image fusion, we combine the direct ptychography and tcDF using Wiener‐type band‐composite weights derived from the per‐channel SSNRs (Methods, Equation (39)).

**FIGURE 2:**
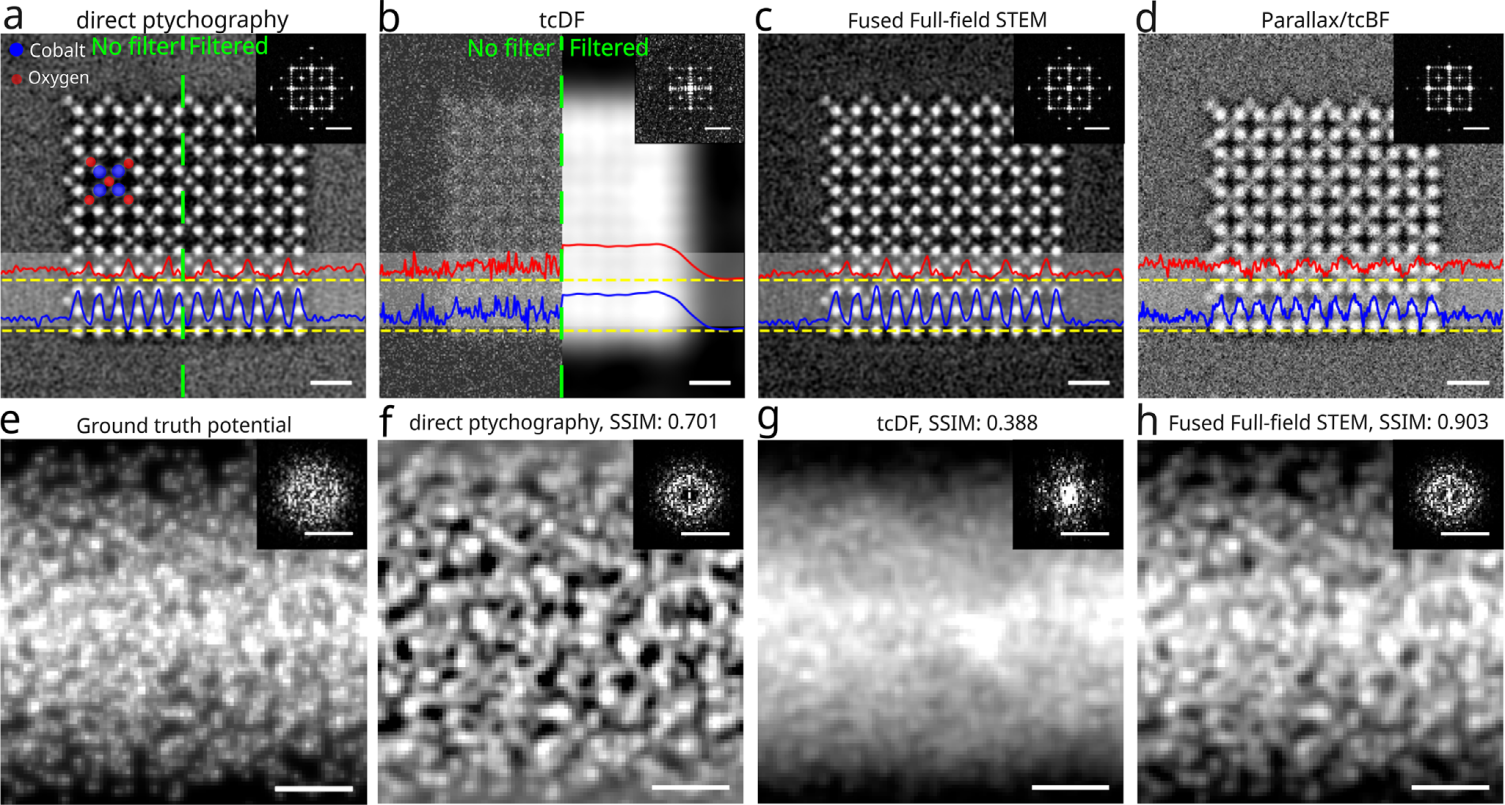
Comparison of ptychography, tcDF, FF‐STEM, and parallax/tcBF reconstructions. (a–d) Atomic‐resolution reconstructions of a ${\mathrm{Co}}_{3}O_{4}$ nanocrystal. In (a), the projected unit cell is indicated. Panels (a) ptychography and (b) tcDF reconstruction. Left half without and the right half with Wiener‐type filters applied. The filtered tcDF (right half of b) shows no atomic contrast because the Wiener weights correctly assign near‐zero weight to tcDF at high spatial frequencies where ptychography has superior SSNR. (c) FF‐STEM reconstruction. (d) Parallax/tcBF reconstruction. (e–h) Reconstructions of a 7.2nm‐thick amorphous carbon wedge. Real‐space scale bars correspond to 5 Å, and reciprocal‐space scale bars correspond to 1 Å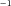.

The direct ptychography result (Figure 2a) clearly resolves atomic columns but exhibits attenuated low‐frequency contrast, leading to reduced background separation between the nanoparticle and vacuum. The tcDF image (Figure 2b), on the other hand, results in significant contrast difference between nanoparticle and vacuum, but with lower spectral signal‐to‐noise at high spatial frequencies compared to the phase‐contrast ptychographic reconstruction. The fused image (Figure 2c) combines the strengths of both methods, producing an improved overall contrast with clearer and less noisy high‐frequency content, as confirmed by the line profiles across the oxygen (red) and cobalt (blue) rows. The FF‐STEM reconstruction preserves the atomic‐resolution detail of the ptychographic channel, as confirmed by the nearly identical high‐frequency line profiles. The enhanced overall contrast between the nanoparticle and the vacuum region reflects the low‐frequency contribution of the tcDF channel. To assess whether FF‐STEM alters the atomic‐column object dependence, we quantified the Co/O column intensity ratio in the simulated ${\mathrm{Co}}_{3}O_{4}$ dataset using local two‐dimensional Gaussian fits with constant background, detailed in (Methods, Equation (47)). The fitted Gaussian peak amplitude was used as the column intensity on several Co and O atom locations. The resulting Co/O ratios were 3.76 ± 0.74 for direct ptychography, 0.97 ± 0.21 for tcDF, 3.50 ± 0.67 for FF‐STEM, and 2.52 ± 0.27 for parallax/tcBF. The close agreement between FF‐STEM and direct ptychography indicates that the fused reconstruction preserves the ptychographic atomic‐column contrast, while the tcDF contribution primarily enhances low‐frequency contrast. Compared with the parallax reconstruction in Figure 2d, the FF‐STEM method achieves comparable atomic resolution with superior background suppression, demonstrating its capability for high contrast aberration‐free imaging in 4D‐STEM.

Figure 2e–h shows a quantitative comparison of a simulated amorphous carbon wedge, where the thickness modulation along the vertical spans the full image. Visually, direct ptychography very weakly transfers this large‐scale contrast variation, as evidenced by the dip in the inset power spectrum at low spatial frequencies, whereas it is excellently preserved in tcDF‐STEM. Quantitatively, we compare the reconstruction fidelity to the simulated ground truth using the structural similarity index measure (SSIM) [33]. Here, FF‐STEM offers the best performance, with a 28.8% improvement in SSIM relative to direct ptychography.

#### 2.2.2 Depth Sectioning From a Single 4D‐STEM Dataset

**FIGURE 3:**
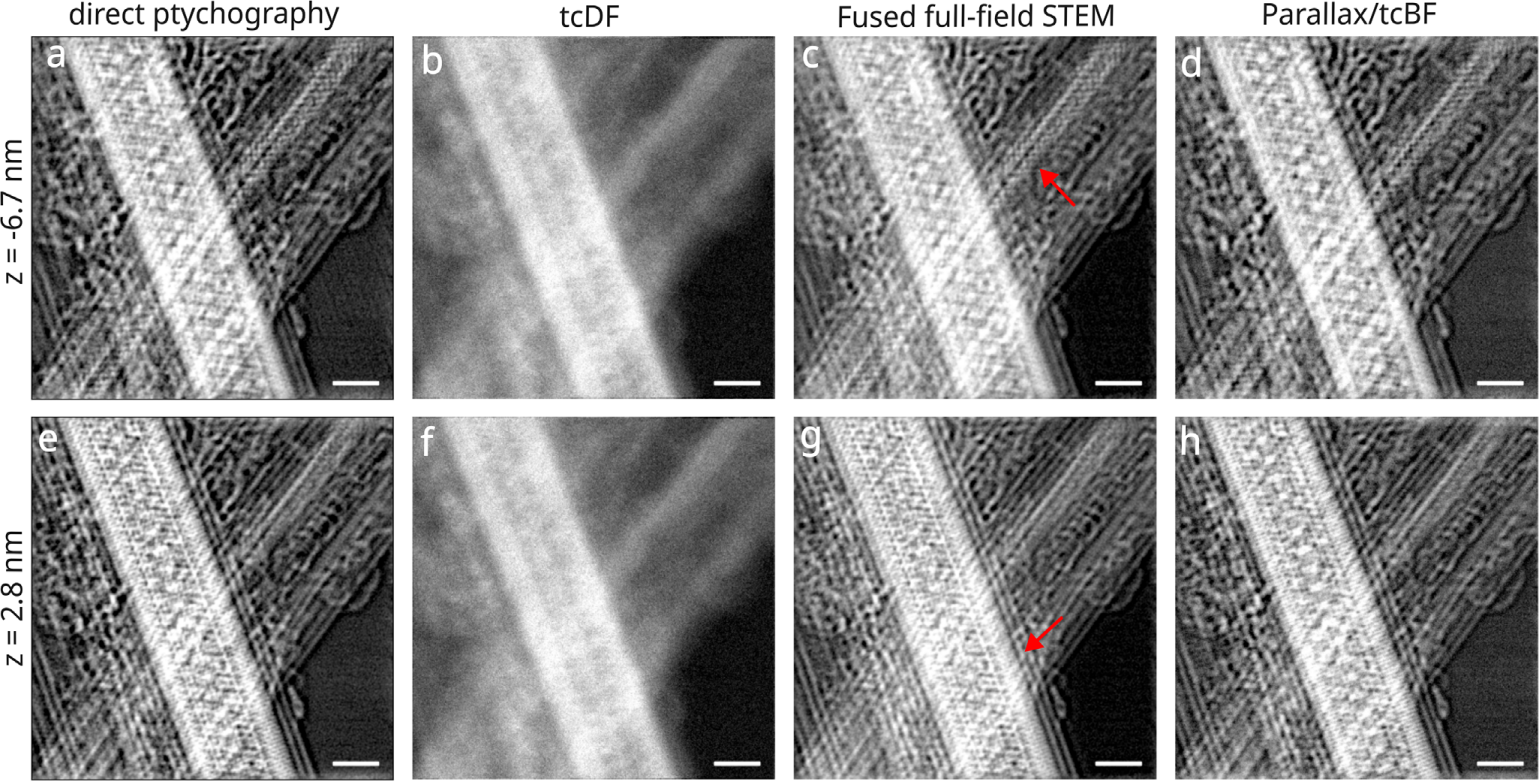
Demonstration of depth‐sectioning capability using FF‐STEM reconstruction. Reconstructed images of Ta–Te core–shell carbon nanotubes with different defocus values. Each column shows results from (a, e) direct ptychography, (b, f) tcDF, (c, g) FF‐STEM, and (d, h) parallax reconstruction. The top row corresponds to a defocus condition in which the reconstruction focuses on the nanotube bundle at the back of the sample, while the bottom row focuses on the front bundle. The parallax reconstruction provides a comparable focal selectivity but lower overall image clarity. Scale bars: 2nm.

To demonstrate the FF‐STEM method's three‐dimensional imaging capability on experimental data, we applied it to a published dataset of a Ta–Te core–shell carbon‐nanotube sample [34]. By systematically varying the defocus value during reconstruction, we obtain depth‐resolved images in which different axial planes of the specimen come into focus. In the upper row of Figure 3, the reconstruction is focused on the nanotube bundle located toward the back of the sample, whereas in the lower row, the focal plane shifts to the front bundle. Among the individual methods, direct ptychography shows distinguishable depth separation between the two focal planes. At the same time, the tcDF reconstructions appear blurred and lack high‐frequency detail, making the focal shift less evident. This is due to the relatively low electron count in the dark field region, which was only recorded up to twice the semiconvergence angle on the 4Dcamera detector. In contrast, the FF‐STEM reconstruction maintains sharp lattice contrast and consistent background noise suppression across depths, indicating robust transfer of information over a broad range of spatial frequencies. The parallax reconstruction shown for comparison produces focal selectivity similar to that shown by direct ptychography. These results confirm that the FF‐STEM approach retains the ability to retrieve 3D information and enables near‐real‐time depth sectioning within a single 4D‐STEM acquisition. We note that this linear depth sectioning does not model multiple scattering, unlike modern nonlinear multi‐slice ptychography (MSP) algorithms [7], but rather provides a near‐real‐time way to gain understanding of the sample geometry and z‐positioning without solving the inverse‐multislice problem. This capability could, for example, be used to initialize MSP reconstructions with reasonable hyperparameters, which typically converge in minutes to tens of minutes with current implementations and single‐node computational resources. A complementary focal‐stack analysis on the diffraction‐grating dataset provides a quantitative estimate of the axial response, yielding a full width at 80% maximum of 4.8 nm (Figure S3).

### 2.3 FF‐STEM for Dose‐Efficient High‐Contrast Imaging in the Materials Sciences

**FIGURE 4:**
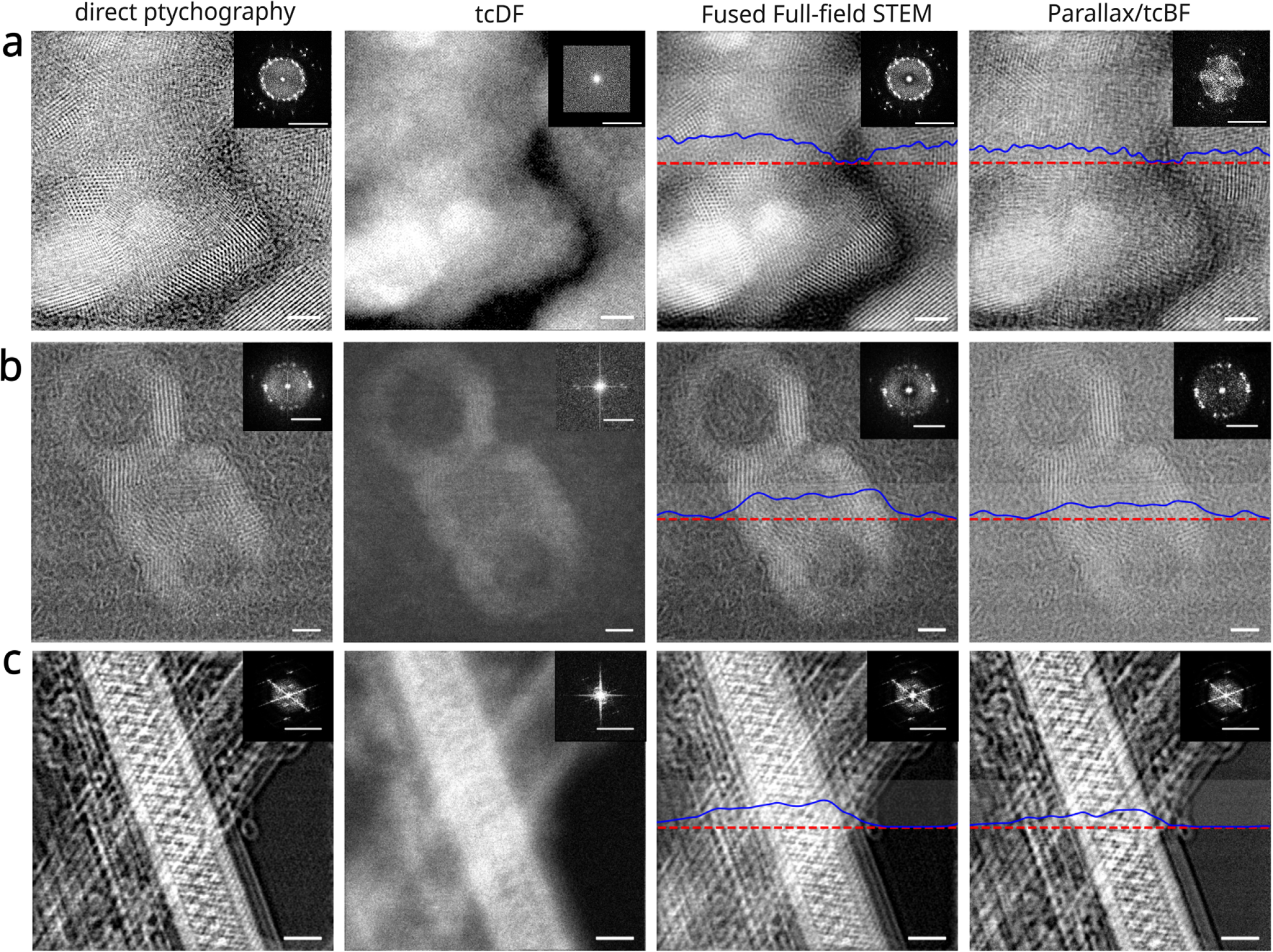
Experimental demonstration of FF‐STEM on various 4D‐STEM datasets. Each row represents a distinct experimental dataset obtained from different materials and detectors. The FF‐STEM results are compared with parallax reconstruction, showing the contrast improvement of the nanoparticle with line profiles overlaid on the images. (a) Reconstructed images of the diffraction grating. All reconstructions, including parallax, are upsampled by a factor of 4. (b) ${\mathrm{Gd}}_{2}O_{3}$ nanohelices acquired with the Timepix4 detector under low dose conditions, all reconstructions are upsampled by 2. (c) Carbon nanotubes with Ta–Te core–shell structure. The red dashed lines in the fused FF and parallax images mark the region used to extract the line profiles (blue curve). The scale bars in real space correspond to 2nm. Insets on the top right show the power spectrum of the corresponding images with reciprocal‐space scale bars of $0.2\;{Å}^{-1}$ for diffraction grating and ${\mathrm{Gd}}_{2}O_{3}$, and $0.5\;{Å}^{-1}$ for Ta–Te.

We further applied FF‐STEM reconstruction to three datasets spanning different materials systems and detector configurations. The first dataset, as shown in Figure 4a, is a standard diffraction‐grating replica generally used for TEM alignment. The dataset is acquired at 200 kV acccelerating voltage, 30 mrad convergence semiangle, 30 pA beam current, and 8 μs dwell time with a 0.727 Å scan step. All reconstructions, direct ptychography, tcDF, FF‐STEM, and parallax are performed with a four‐fold upsampling to the Nyquist limit, with tcDF and parallax upsampled by zero‐padding in Fourier space, and direct ptychography upsampled by tiling in Fourier space, explained in Methods (Equation (7)). In the upsampled reconstructions, the FF‐STEM image exhibits enhanced lattice continuity and smoother contrast modulation, accompanied by reduced background noise in both real and reciprocal space. The upsampled parallax reconstruction, while capturing the overall periodicity, shows relatively noisy background. The corresponding power spectra show that the FF‐STEM image recovers more Bragg peaks and exhibits a lower diffuse background intensity. The line profile taken along the red dashed region in the FF‐STEM image further illustrates the improved signal‐to‐noise ratio achieved by the FF‐STEM approach. Additionally, a dose series acquired on the diffraction‐grating replica further shows that the SSNR‐based fusion adapts to electron dose, progressively reducing the tcDF contribution as the dark‐field channel becomes noise‐limited at lower fluence (Figure S1).

The second dataset in Figure 4b, showing ${\mathrm{Gd}}_{2}O_{3}$ [35] nanohelices, is recorded at 60 keV electron energy and a 30 mrad semiconvergence angle using a Timepix4 detector at a fluence of $1211e/{Å}^{2}$. It showcases the method's robustness under low‐count and very fast dwell‐time conditions of a modern event‐based detector using 1 μs dwell time and 36pA beam current. With an upsampling factor of 2, the reconstructed image clearly resolves atomic features at a Nyquist resolution of 0.86 Å, while the corresponding power spectrum inset reveals distinct Bragg reflections and reduced low‐frequency background, confirming that the fusion suppresses the noise from low‐count binary datasets [36]. Such event‐based datasets recorded with ultrafast dwell times can be beneficial for very low‐dose imaging of beam‐sensitive materials [37], and might enable motion correction for cryo‐electron ptychography [38].

Finally, we applied FF‐STEM on a different region of Ta–Te core–shell carbon nanotubes [34], acquired under multiple‐scattering conditions with the 4Dcamera at 80 keV and 25mrad semi‐convergence angle. The FF‐STEM reconstruction enhances both the high‐frequency periodicity of the Ta–Te lattice and the low‐frequency envelope contrast of the surrounding carbon walls. Across all four experiments, the fused method consistently delivers balanced low‐ and high‐frequency transfer, confirming its generalization beyond simulations and its suitability for high‐contrast imaging in diverse experimental settings. The dependence on detector angular range is further examined in Figure S5, which shows that restricting the collected dark‐field scattering angle suppresses the tcDF SSNR and reduces the low‐frequency enhancement in the fused FF‐STEM reconstruction.

### 2.4 FF‐STEM for Dose‐Efficient Imaging of Thick Samples in Cryo‐Electron Microscopy

**FIGURE 5:**
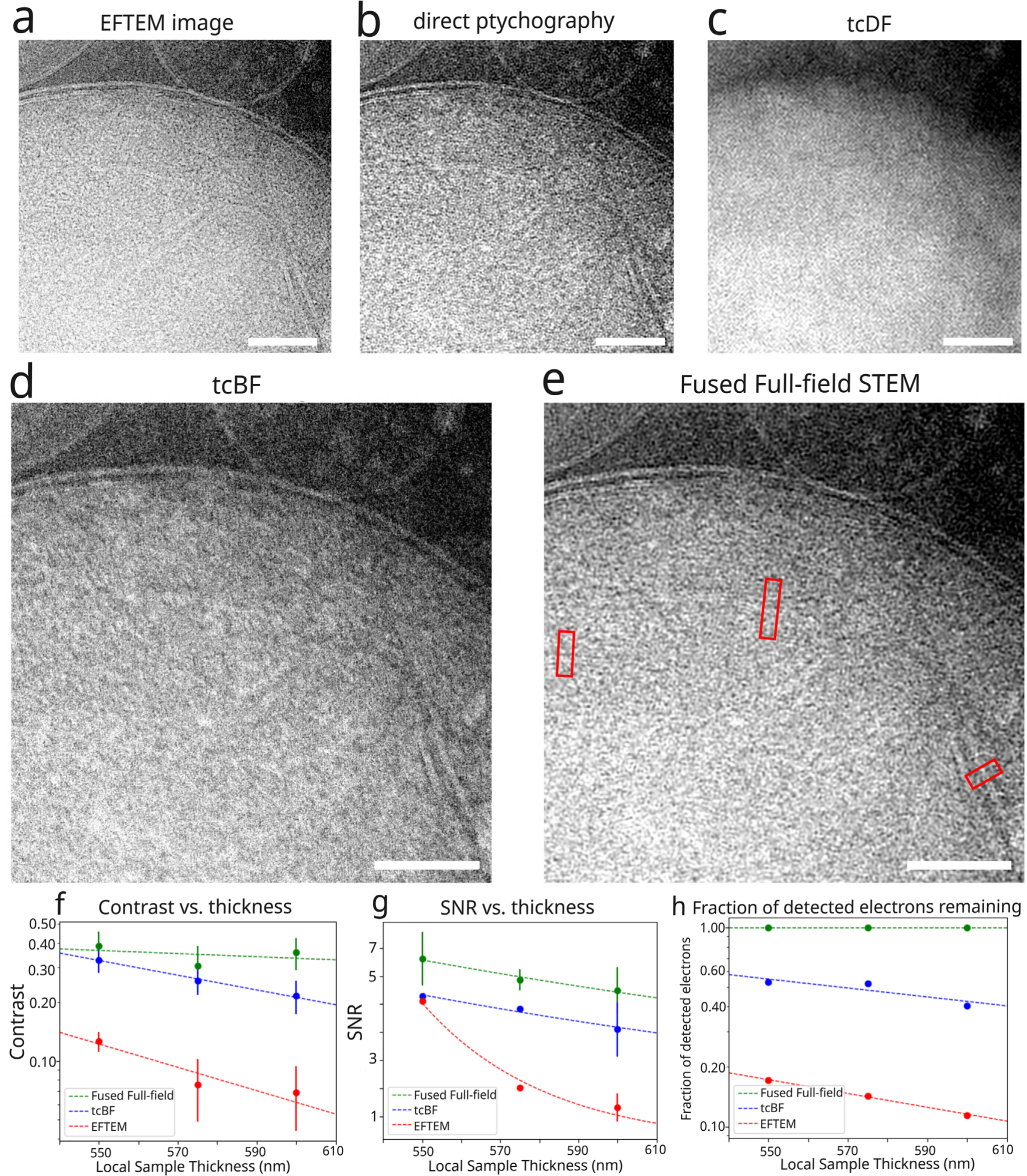
Dose‐efficient cryogenic imaging of thick specimens using FF‐STEM. (a) EFTEM image reproduced from [22], acquired at $14e/{Å}^{2}$. (b) Direct ptychography reconstruction from the 4D‐STEM dataset (c) tcDF reconstruction, (d) tcBF image reproduced from [22], (e) FF‐STEM reconstruction. Red rectangles indicate regions of interest used for quantitative analysis. (f) Image contrast, (g) signal‐to‐noise ratio (SNR), and (h) fraction of detected electrons remaining, all extracted from identical regions of interest marked in panel (e). Error bars represent the root‐mean‐square variation of the measured line profiles. The scale bar is 100nm.

As a second application, we evaluate FF‐STEM for imaging of thick biological materials. Figure 5 compares energy‐filtered TEM and various 4D‐STEM imaging methods on a published dataset, imaging an approximately 600 nm thick region of a mitochondrion at 300kV, collected at a fluence of $14e/{Å}^{2}$ [22]. As in the original publication, we compare the fringe contrast and signal‐to‐noise ratio of the membrane bilayer features, as well as the fraction of detected electrons used in the imaging method.

As in the original publication [22], tcBF imaging shows improved contrast compared to EFTEM, and the fraction of electrons contributing to the image is dramatically increased. Direct ptychography provides similar, but slightly better contrast than tcBF, which was recently also explained from an SSNR point of view [19, 29, 30]. This contrast improvement is carried over to FF‐STEM, which also inherits the thickness and low spatial frequency contrast from the tcDF signal. A direct side‐by‐side comparison with identical contrast normalization further confirms that FF‐STEM preserves the membrane‐like features visible in direct ptychography while improving sample–background separation (Figure S6).

FF‐STEM consistently exhibits higher contrast and SNR while retaining a substantially larger fraction of detected electrons than tcBF and EFTEM across the full thickness range, reflecting its ability to make use of electrons scattered into both the bright‐field and dark‐field regions of the detector.

**FIGURE 6:**
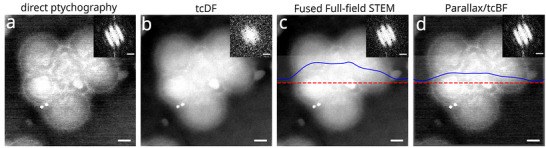
Thickness‐ and phase‐contrast at low fluence and high convergence angles. Vitrified Virus‐like particle cluster imaged under cryogenic conditions with a fluence of $32e/{Å}^{2}$ and 30 mrad convergence angle. Insets on the top right show the power spectrum of the corresponding images. The scale bar in real space is 40nm. in reciprocal‐space is 0.1$\;{Å}^{-1}$.

The last dataset shown in Figure 6 demonstrates FF‐STEM imaging of a virus‐like particle (VLP) and is recorded at 200 keV electron energy and a 30 mrad semiconvergence angle using a Dectris ARINA detector with 10μs dwell time at a fluence of $32e/{Å}^{2}$, highlighting the method's capability to recover mesoscale structure with strong low‐frequency components. The reconstructed image preserves the particle morphology and fine structural variations despite the relatively large field of view, demonstrating the method's adaptability across length scales. Large convergence angles allow depth resolution of a few nanometers, but such high convergence angles strongly dampen large‐scale features across the whole virus particle in ptychographic reconstructions. These features are preserved in the dark‐field signal and subsequently in FF‐STEM. They can be used in the future to provide excellent depth information in limited‐angle tomography experiments, similar to recent approaches that exploit so‐called shadow montages [39] to extract 3D information from single 4D‐STEM datasets acquired for tomography. The vitrified virus‐like particle cluster represents a substantially thinner biological specimen than the mitochondrion dataset, although its local thickness was not independently quantified.

## 3 Discussion

In summary, we have developed the Fused Full‐field 4D‐STEM method that combines the complementary strengths of direct ptychography and tcDF imaging within a single, analytical framework. The approach simultaneously enhances low‐ and high‐spatial‐frequency contrast, enables automated aberration determination and compensation, and supports Fourier‐domain upsampling and depth sectioning, all while maintaining near‐real‐time performance. Through simulations and multiple experimental datasets spanning nanocrystalline oxides, carbon nanotubes, and thick biological samples, FF‐STEM yields high‐contrast images across diverse materials systems and detector configurations. The method preserves atomic‐resolution detail, reveals specimen depth information, and achieves complete reconstruction of multi‐gigabyte 4D‐STEM data within half a second with current parallel processing hardware.

These results establish FF‐STEM as a versatile, computationally efficient tool for high‐contrast, highly dose‐efficient STEM imaging and open the way for near‐real‐time feedback and adaptive microscopy in complex electron‐scattering experiments.

## 4 Methods

### 4.1 Upsampled Direct Ptychography

Here, k denotes the detector‐plane (diffraction) coordinate and q denotes the specimen‐plane spatial frequency, conjugate to the scan coordinate r. The Fourier transform of a 4D‐STEM dataset in the projection approximation can be described as the function G(k,q) [40]

(1) $$\begin{array}{ccc} G(k,q) & = & {\left|A\left(k\right)\right|}^{2}\delta \left(q\right)+A\left(k\right)A^{*}\left(k+q\right)O^{*}\left(-q\right)\\ & & +\;A^{*}\left(k\right)A\left(k-q\right)O\left(+q\right). \end{array}$$

Here we use the Fourier‐space complex aperture function

(2) $$A\left(k\right)\;=\;H\left(k\right)\;e^{-\;i\;\chi (k)},$$

where H(k) is the aperture amplitude (top‐hat in the implementation) and χ(k) is the aberration phase. Using the weak‐phase approximation, it can be shown that

(3) $$G\left(k,q\right)={\left|A\left(k\right)\right|}^{2}\delta \left(q\right)+\Gamma \left(k,q\right)O\left(q\right)$$

where Γ is the so‐called aperture‐overlap function:

(4) $$\begin{array}{ccc} \Gamma (k,q) & = & A\left(k\right)A\left(q-k\right)e^{-i\chi (q-k)}e^{i\chi (k)}\\ & & -\;A\left(k\right)A\left(q+k\right)e^{i\chi (q+k)}e^{-i\chi (k)} \end{array}$$

If the aberrations are fixed and known, a bright‐field phase‐contrast image can then be calculated directly from G(k,q):

(5) $$O\left(r\right)=ℑ\left[F_{q\to r}^{-1}\left[\sum_{k}G\left(k,q\right)\frac{\Gamma^{*}\left(k,q\right)}{\left|\Gamma (k,q)\right|}\right]\right],\;\text{for}\;k\in \left\{|A\left(k\right)|\neq 0\right\}.$$

This matched‐filter reconstruction appeared first in [40] without an explicit name. In recent works, it has been named “direct ptychography” [41], “SSB+TO” [30] and “acBF‐STEM” [19] with notational differences and equivalence up to 90 degree rotation in the complex plane. Recently, the possibility of upscaling the reconstructed image beyond the used scan step was introduced by either tiling in Fourier space [41] or interleaving zeros in real‐space [22]. Here we use the approach by [41] as it reduces the computational complexity of the reconstruction.

(6) $$G^{\prime }\left(k,q\right)={\mathrm{tile}}_{f}\left[G(k,q)\right]$$

The final weak‐phase image is then computed with upsampled coordinates using summation over the bright‐field pixels and correcting aberrations first by multiplying with the aperture overlap function Γ

(7) $$\begin{array}{ccc} O(r) & = & ℑ\left[F_{q\to r}^{-1}\left[\sum_{k}G^{\prime }\left(k,q\right)\frac{\Gamma^{*}\left(k,q\right)}{\left|\Gamma (k,q)\right|}\right]\right],\\ & & \text{for}\;k\in \{|\Gamma (k,q)|\neq 0\}. \end{array}$$

Contrast in ptychography is strongly influenced by aberrations, with defocus being the simplest way to improve transfer of low spatial frequencies. This has been discussed in detail in excellent recent works [19, 24, 41]. Spherical aberration delivers a contrast advantage for ptychography [24, 42]. Using an uncorrected microscope with a defocused probe and spherical aberration therefore offers contrast advantages. For ease of use in aberration corrected microscopes, defocus alone suffices. Figure S2 shows the influence of defocus on the SSNR using simulated data.

### 4.2 Aberration Determination Through Backpropagation of Image‐Contrast Gradients

We find the aberrations by maximizing an image contrast metric and backpropagating the gradients of this metric through the direct ptychography reconstruction to the aberration coefficients. Namely, we optimize the Total Variation loss function with respect to the 12 Cartesian aberration coefficients parameterizing aberrations up to and including third order in scattering angle.

(8) $${\hat{a}}_{1\text{…}12}=\arg\min_{a_{1\text{…}12}}\left(-\;\mathrm{TV}(O(r)\right)$$

This approach is known to work also in the visible light regime [43], albeit using an iterative reconstruction algorithm.

### 4.3 Analytical Gradients of the Ptychographic Aberration Parameters

In our implementation, we use Cartesian aberration coefficients [44] to avoid evaluating transcendental functions. With $u=k_{x}\lambda$, $v=k_{y}\lambda$, we define the aberration function

$$\chi \left(k\right)=\frac{2\pi }{\lambda }\sum_{j=0}^{11}a_{j}\;\phi_{j}\left(u,v\right),$$

with the basis functions $\phi_{j}\left(u,v\right)$

$$\begin{array}{ccc} \phi_{0} & & =\frac{1}{2}\left(u^{2}+v^{2}\right),\;\phi_{1}=\frac{1}{2}\left(u^{2}-v^{2}\right),\\ \phi_{2} & & =uv,\\ \phi_{3} & & =\frac{1}{3}\left(u^{3}+uv^{2}\right),\;\phi_{4}=\frac{1}{3}\left(v^{3}+u^{2}v\right),\\ \phi_{5} & & =\frac{1}{3}\left(u^{3}-3uv^{2}\right),\;\phi_{6}=\frac{1}{3}\left(3u^{2}v-v^{3}\right),\\ \phi_{7} & & =\frac{1}{4}\left(u^{4}+v^{4}+2u^{2}v^{2}\right),\;\phi_{8}=\frac{1}{4}\left(u^{4}-v^{4}\right),\\ \phi_{9} & & =\frac{1}{4}\left(2u^{3}v+2uv^{3}\right),\;\phi_{10}=\frac{1}{4}\left(u^{4}-6u^{2}v^{2}+v^{4}\right),\\ \phi_{11} & & =\frac{1}{4}\left(4u^{3}v-4uv^{3}\right). \end{array}$$

where we map the coefficients $a_{j}$ to the known aberration coefficients in the following way: $a_{0}=C_{1}$, $a_{1}=C_{1,2a}$, $a_{2}=C_{1,2b}$, $a_{3}=C_{2,1a}$, $a_{4}=C_{2,1b}$, $a_{5}=C_{2,3a}$, $a_{6}=C_{2,3b}$, $a_{7}=C_{3}$, $a_{8}=C_{3,2a}$, $a_{9}=C_{3,2b}$, $a_{10}=C_{3,4a}$, $a_{11}=C_{3,4b}$. Therefore

(9) $$\frac{\partial \chi (k)}{\partial a_{j}}\;=\;\frac{2\pi }{\lambda }\;\phi_{j}\left(u,v\right).$$

Given a real loss L with upstream adjoint $\Delta \left(q,k\right)=\partial L/\partial G_{\text{out}}\left(q,k\right)$, with $G_{\text{out}}$ being the aberration‐corrected G‐function, for any real parameter p,

(10) $$\frac{\partial L}{\partial p}=\sum_{q,k}ℜ\;\left\{\bar{\Delta (q,k)}\;\frac{\partial G_{\text{out}}\left(q,k\right)}{\partial p}\right\}=\sum_{q,k}ℜ\;\left\{\bar{\Delta }\;G_{\text{in}}\;\bar{\frac{\partial \Gamma }{\partial p}}\right\},$$

with $G_{\text{in}}$ the uncorrected G‐function. The derivative $\frac{\partial \Gamma }{\partial p}$ is given in the supplementary materials. Combining Equations (9) and $\frac{\partial \Gamma }{\partial p}$ (13) yields

(11) $$\begin{array}{ccc} \frac{\partial L}{\partial a_{j}} & = & \sum_{q,k}ℜ\;\left\{\bar{\Delta (q,k)}\;\right.\\ & & \left.G_{\text{in}}\left(q,k\right)\;\bar{\;i\;\left[C_{-}\left(\partial \chi_{0}-\partial \chi_{-}\right)+C_{+}\left(\partial \chi_{0}-\partial \chi_{+}\right)\right]}\right\}, \end{array}$$

where $C_{-}=\bar{A(k)}\;A\left(k-q\right)$ and $C_{+}=A\left(k\right)\;\bar{A(k+q)}$, and $\partial \chi_{\left\{\cdot \right\}}=\partial \chi \left(k\left\{\cdot \right\}\right)/\partial a_{j}$ from Equation (3).

Here, we use a hard aperture, which simplifies gradient calculation. For a soft aperture, product‐rule terms proportional to $\partial H/\partial a_{j}$ at k, k±q must be included. In our implementation: complex i is the $90^{\circ }$ rotation (x,y)↦(−y,x) on the $R^{2}$ view of C. Accumulation uses only the real parts $ℜ\{\bar{\Delta }\;\cdot \}$. We implement this gradient calculation with efficient customized CUDA kernels. The main implementation challenge is the summation over both q and k, which is inefficient when implemented with a trivial atomic addition on massively parallel architectures. Here we opt for a tile‐wise sum, where each core performs summation over it's (q,k) components before the global summation.

### 4.4 Choice of SSNR as the Information Metric

We use the spectral signal‐to‐noise ratio (SSNR) rather than the contrast transfer function (CTF) as the primary metric because the SSNR captures both the deterministic transfer characteristics and the noise level at each spatial frequency. The CTF describes only the signal modulation without accounting for noise, and is therefore insufficient for optimal data fusion where the reliability of each channel must be quantified. The SSNR‐based Wiener weights provide the minimum‐noise‐variance band composite of the two channels under the band‐passthrough constraint (see “Linear fusion: FF‐STEM as a Wiener band composite”), which is not achievable with CTF‐based weighting alone.

### 4.5 Analytical SSNR of Direct Ptychography

Under the weak phase object approximation (WPOA), the ptychographic contrast transfer function is specimen‐independent and depends only on the probe wavefunction

(12) $$\psi \left(k\right)=A\left(k\right)\;e^{-i\chi (k)},$$

where A(k) is the normalized top‐hat aperture and χ(k) is the aberration phase parameterized by Seidel coefficients.

Following Refs. [29, 30], the dose‐normalized spectral signal‐to‐noise ratio (SSNR) is

(13) $${\mathrm{SSNR}}_{\mathrm{ptycho}}\left(q\right)=\frac{\sum_{k}\left|\psi^{*}\left(k\right)\psi \left(q-k\right)-\psi \left(k\right)\psi^{*}\left(q+k\right)\right|}{2\;\sqrt{N_{\mathrm{DO}+\mathrm{TO}}\left(q\right)}},$$

where $N_{\mathrm{DO}+\mathrm{TO}}\left(q\right)$ is the number of pixels in the double‐overlap and triple‐overlap regions of the aperture autocorrelation at spatial frequency q.

### 4.6 Tilt‐Corrected Dark‐Field STEM

Tilt‐corrected dark‐field STEM was introduced by Ma et al. [19]. Starting from Equation (8) of [26], the usual ADF incoherent model $I\left(r\right)={\left|P\right|}^{2}*O\left(r\right)$ follows when the detector integral becomes independent of the incident angles over the aperture‐overlap domain, that is, for a geometrically large annular detector. A small dark‐field sector violates this condition, so the corresponding $I_{m}\left(r\right)$ retains coherent dependencies on $\left(\theta ,\theta^{\prime }\right)$ and does not reduce to a simple intensity convolution. In the 4D‐STEM contrast decomposition of [19], however, only the incoherent amplitude term contributes in the dark‐field region. A practical tcDF image is obtained by azimuthally shifting each dark‐field sector by $\Delta \rho_{m}=\left(\Delta f+\Delta z\right)\left(2\alpha /3\right)u_{\varphi_{m}}$ and then summing over all sectors, which preferentially aggregates this incoherent channel while restoring the bright‐field‐like parallax. The resulting tcDF image therefore shows partially coherent amplitude contrast and depth‐sectioning behavior. It approaches the ADF‐like ${\left|P\right|}^{2}$ convolution only when the summed sector coverage effectively behaves like a large annulus, or when the scattering is sufficiently localized to render the detector integral effectively independent of the incident angles. The following steps describe the practical procedure to compute $I_{\mathrm{tcDF}}$ from a 4D‐STEM dataset (Algorithm 1).

ALGORITHM 1 **Computation of the tcDF‐STEM image from 4D‐STEM data.**

**Table:** 

**Require**: 4D dataset I(r,Θ)	
**Require**: detector angle maps $\left(\Theta_{x},\Theta_{y}\right)$	
**Require**: convergence semi‐angle α	
**Require**: DF annulus $\left[\beta_{\min},\beta_{\max}\right]$	
**Require**: defocus Δf	
**Require**: depth offset Δz	
**Require**: scan pixel size $s_{\mathrm{scan}}$	
1:	**Partition the detector**. Divide the dark‐field region into M narrow angular sectors of width Δφ=2π/M: (14) $$D_{m}\left(\Theta \right)=W_{\mathrm{DF}}\left(|\Theta |\right)\;\Pi_{\Delta \varphi }\;\left(\arg\Theta -\varphi_{m}\right),$$ with $W_{\mathrm{DF}}=1$ for $\beta_{\min}\;\leq \left|\Theta \right|\;\leq \beta_{\max}$ and 0 otherwise.
2:	**Integrate each sector**. For every sector m, integrate the 4D intensity over the detector pixels belonging to that sector to obtain a real‐space image (15) $I_{m}\left(r\right)=\int D_{m}\left(\Theta \right)\;I\left(r,\Theta \right)\;d\Theta .$
3:	**Compute the tcDF parallax shift**. For each sector m with azimuth $\varphi_{m}$, calculate the shift vector (16) $$\begin{array}{c} \Delta \rho_{m}=\left(\Delta f+\Delta z\right)\;\theta_{\mathrm{ref}}\left(\varphi_{m}\right),\;\theta_{\mathrm{ref}}\left(\varphi_{m}\right)=\frac{2\alpha }{3}\;u_{\varphi_{m}}, \end{array}$$ and convert it to scan‐pixel units, $\Delta p_{m}=\Delta \rho_{m}/s_{\mathrm{scan}}$.
4:	**Apply the real‐space shift**. Shift each sector image $I_{m}\left(r\right)$ by $\Delta p_{m}$ in scan coordinates. We use a Fourier‐domain shift: (17) $$I_{m}^{\mathrm{shifted}}\left(r\right)=F^{-1}\;\left\{F\;\left[I_{m}\left(r\right)\right]e^{-i2\pi Q\cdot \Delta \rho_{m}}\right\}.$$
5:	**Sum the shifted sectors**. Sum all shifted sector images to form the tcDF image: (18) $O_{\mathrm{tcDF}}\left(r\right)=\sum_{m=1}^{M}I_{m}^{\mathrm{shifted}}\left(r\right).$
6:	**Output**: The tcDF image $O_{\mathrm{tcDF}}\left(r\right)$, which exhibits amplitude‐dominated (incoherent) contrast and enables defocus‐ or height‐dependent depth sectioning through the choice of (Δf+Δz).

The number M of detector segments is a free parameter and we determined M=32 as a reasonable tradeoff between runtime and shift precision by manual search.

### 4.7 Half‐Data SSNR Estimation for tcDF‐STEM

The tcDF‐STEM signal depends on object‐dependent elastic scattering amplitudes, and no closed‐form SSNR exists; we therefore estimate ${\mathrm{SSNR}}_{\mathrm{tcDF}}$ using two statistically independent half‐data reconstructions.

The dark‐field detector is divided azimuthally. We create two independent reconstructions by assigning alternating DF segments to two groups, reconstructing two tcDF images using the identical algorithm. Let

(19) $$F_{A}\left(q\right),\;F_{B}\left(q\right)$$

denote the orthonormal Fourier transforms of the two half‐data images.

We define half‐sum and half‐difference spectra

(20) $$S\left(q\right)=\frac{1}{2}\left(F_{A}\left(q\right)+F_{B}\left(q\right)\right),\;N\left(q\right)=\frac{1}{2}\left(F_{A}\left(q\right)-F_{B}\left(q\right)\right).$$

The radially averaged noise and total power spectra are

(21) $$P_{\mathrm{noise}}\left(q\right)={\langle {\left|N\left(q\right)\right|}^{2}\rangle }_{|q|=q},$$

(22) $$P_{\mathrm{total}}\left(q\right)={\langle {\left|S\left(q\right)\right|}^{2}\rangle }_{|q|=q}.$$

The signal power is obtained by noise subtraction,

(23) $$P_{\mathrm{signal}}\left(q\right)=P_{\mathrm{total}}\left(q\right)-P_{\mathrm{noise}}\left(q\right),\;P_{\mathrm{signal}}\left(q\right)\geq 0.$$

The tcDF spectral signal‐to‐noise ratio is

(24) $${\mathrm{SSNR}}_{\mathrm{tcDF}}\left(q\right)=\frac{P_{\mathrm{signal}}\left(q\right)}{P_{\mathrm{noise}}\left(q\right)}.$$

### 4.8 Linear Fusion: FF‐STEM as a Wiener Band Composite

The FF‐STEM image is constructed as a frequency‐dependent Wiener band composite of the direct‐ptychography and tcDF reconstructions. As detailed in the main text and Discussion, and as follows from the contrast decomposition of [19] (Equation 7), the bright‐field and dark‐field regions of a 4D‐STEM dataset carry different contrast functionals of the specimen — coherent phase ∝σV in the BF region and incoherent amplitude $\propto \sigma^{2}V^{2}$ in the DF region. The two channels are therefore intentionally complementary, and the fusion combines them in the band where each is maximally reliable.

At each spatial frequency q we model

(25) $$F_{i}\left(q\right)=S_{i}\left(q\right)+n_{i}\left(q\right),\;i\in \left\{\mathrm{ptycho},\;\mathrm{tcDF}\right\},$$

where $S_{i}\left(q\right)$ is the (unknown) noise‐free reconstruction of channel i — a deterministic functional of the specimen potential whose explicit form we do not require — and $n_{i}\left(q\right)$ is a zero‐mean noise term with

(26) $$E\left[n_{i}\left(q\right)\right]=0,\;E\left[|n_{i}{\left(q\right)|}^{2}\right]=\sigma_{i}^{2}\left(q\right),$$

and $n_{\mathrm{ptycho}}\left(q\right)$, $n_{\mathrm{tcDF}}\left(q\right)$ uncorrelated for each q. The independence of the noise terms is justified by the fact that the two channels are formed from disjoint subsets of the diffraction data (BF disk vs. DF region) and that the dominant detector noise is shot noise, uncorrelated between non‐overlapping pixels.

We seek a linear fused estimator of the form

(27) $$\hat{F}\left(q\right)=w_{1}\left(q\right)\;F_{1}\left(q\right)+w_{2}\left(q\right)\;F_{2}\left(q\right),\;w_{1}\left(q\right)+w_{2}\left(q\right)=1.$$

The constraint $w_{1}+w_{2}=1$ is a *band‐passthrough* condition: in any frequency band where one channel is overwhelmingly more reliable than the other ($w_{i}\to 1$), the fused output passes that channel's signal through unchanged. The signal of the dominant channel in each band is preserved, while the contribution of the noisier channel is suppressed.

#### 4.8.1 Gaussian Noise Model

Under the above assumptions the noise contribution to the fused estimator is

(28) $$\hat{F}\left(q\right)-\left(w_{1}S_{1}+w_{2}S_{2}\right)\left(q\right)=w_{1}\left(q\right)\;n_{1}\left(q\right)+w_{2}\left(q\right)\;n_{2}\left(q\right),$$

with variance

(29) $${\mathrm{Var}}_{\mathrm{noise}}\left[\hat{F}\left(q\right)\right]=|w_{1}{\left(q\right)|}^{2}\sigma_{1}^{2}\left(q\right)+{\left|w_{2}\left(q\right)\right|}^{2}\sigma_{2}^{2}\left(q\right),$$

because the cross‐term vanishes for uncorrelated noise. Using $w_{2}\left(q\right)=1-w_{1}\left(q\right)$, we minimize this noise variance with respect to $w_{1}\left(q\right)$:

(30) $$\frac{\partial }{\partial w_{1}}\;\left[|w_{1}{|}^{2}\sigma_{1}^{2}+{\left|1-w_{1}\right|}^{2}\sigma_{2}^{2}\right]=2w_{1}\sigma_{1}^{2}-2\left(1-w_{1}\right)\sigma_{2}^{2}=0,$$

which yields the familiar inverse‐variance weights

(31) $$w_{1}\left(q\right)=\frac{\sigma_{2}^{-2}\left(q\right)}{\sigma_{1}^{-2}\left(q\right)+\sigma_{2}^{-2}\left(q\right)},\;w_{2}\left(q\right)=\frac{\sigma_{1}^{-2}\left(q\right)}{\sigma_{1}^{-2}\left(q\right)+\sigma_{2}^{-2}\left(q\right)}.$$

These are the unique weights that minimize the noise variance of $\hat{F}\left(q\right)$ subject to the band‐passthrough constraint $w_{1}+w_{2}=1$. They are formally identical to the Gauss–Markov estimator of a common signal under uncorrelated Gaussian noise; we recover the same expression here, but with the interpretation of a noise‐minimizing band composite of two channels whose noise‐free signals $S_{1}\left(q\right)\neq S_{2}\left(q\right)$ in general.

#### 4.8.2 Formulation in Terms of SSNR

For each channel, define the spectral signal‐to‐noise ratio relative to that channel's noise‐free signal power $S_{i,\mathrm{pow}}\left(q\right)=E\left[|S_{i}{\left(q\right)|}^{2}\right]$:

(32) $${\mathrm{SSNR}}_{i}\left(q\right)=\frac{S_{i,\mathrm{pow}}\left(q\right)}{\sigma_{i}^{2}\left(q\right)}.$$

For the ptychography channel, ${\mathrm{SSNR}}_{\mathrm{ptycho}}\left(q\right)$ follows an analytical form derived from the aperture‐overlap autocorrelation [29, 30] (Equation (13)). For the tcDF channel, no analytical SSNR is available because the signal is object‐dependent [19, 28]; it is therefore estimated empirically from two independent half‐data tcDF reconstructions using the half‐split estimator from cryo‐EM [31, 32] (Equation (24)).

In the special case where the two channels happened to estimate the same signal with $S_{1,\mathrm{pow}}\left(q\right)=S_{2,\mathrm{pow}}\left(q\right)=S_{X}\left(q\right)$, that common factor would cancel between the inverse‐variance weights and one would recover the symmetric SSNR weights

(33) $$w_{i}\left(q\right)=\frac{{\mathrm{SSNR}}_{i}\left(q\right)}{\sum_{j}{\mathrm{SSNR}}_{j}\left(q\right)}.$$

In FF‐STEM this special case does not hold rigorously, because the two channels carry different contrast functionals of the specimen. The weights in Equation (33) are nevertheless adopted: they assign trust at each spatial frequency in proportion to per‐channel reliability, and at frequencies where one SSNR vanishes they automatically select the other channel. The fused image is therefore best understood as a Wiener band composite that suppresses each channel's contribution where its own SSNR is small, rather than as an unbiased estimator of a shared object.

#### 4.8.3 Extension to Poisson Noise

Many electron microscopes are nowadays equipped with direct electron detectors, where the dominant noise source is Poisson‐distributed counting noise. Consider, for simplicity, a single scalar degree of freedom (e.g. one pixel or one Fourier coefficient) measured twice with different modalities and electron doses $g_{1}$ and $g_{2}$. The measured counts $y_{i}$ obey

(34) $$y_{i}∼\mathrm{Poisson}\left(\lambda_{i}\right),\;\lambda_{i}=g_{i}\;s_{i},$$

where $s_{i}$ is the underlying (dose‐normalized) channel signal — in general different across channels, since they carry different contrast functionals. A dose‐normalized estimator of channel i is

(35) $$X_{i}=\frac{y_{i}}{g_{i}},\;E\left[X_{i}\right]=s_{i},$$

with variance

(36) $$\mathrm{Var}\left(X_{i}\right)=\frac{s_{i}}{g_{i}}.$$

Minimizing the noise variance of the band composite $\hat{s}=w_{1}X_{1}+w_{2}X_{2}$ under $w_{1}+w_{2}=1$ yields the same inverse‐variance form as in the Gaussian case, with $\sigma_{i}^{2}\to s_{i}/g_{i}$. More generally, any linear transformation of Poisson counts (e.g. formation of complex Fourier coefficients or reconstructed images) yields, by the central limit theorem, approximately Gaussian noise with a variance that can be computed from the underlying Poisson statistics. Once $\sigma_{i}^{2}\left(q\right)$ of each reconstruction is known or estimated, the above derivation applies unchanged. The Wiener band composite in the presence of Poisson noise is therefore still achieved by inverse‐variance weighting,

(37) $$w_{i}\left(q\right)\propto \frac{1}{\sigma_{i}^{2}\left(q\right)},$$

or, equivalently, by SSNR weighting,

(38) $$w_{i}\left(q\right)=\frac{{\mathrm{SSNR}}_{i}\left(q\right)}{\sum_{j}{\mathrm{SSNR}}_{j}\left(q\right)}.$$

In this sense, the SSNR‐based weighting provides the minimum‐noise‐variance Wiener band composite of multiple independent reconstructions, both under Gaussian additive noise and when the noise originates from Poisson counting statistics.

### 4.9 Wiener‐Type Fusion Weights

For two channels with independent noise and per‐channel SSNRs ${\mathrm{SSNR}}_{1}\left(q\right)$ and ${\mathrm{SSNR}}_{2}\left(q\right)$, the minimum‐noise‐variance Wiener band composite under the band‐passthrough constraint $w_{1}+w_{2}=1$ assigns the weights given in Equation (33). The FF‐STEM weights are therefore

(39) $$\begin{array}{ccc} w_{\mathrm{ptycho}}\left(q\right) & = & \frac{{\mathrm{SSNR}}_{\mathrm{ptycho}}\left(q\right)}{{\mathrm{SSNR}}_{\mathrm{ptycho}}\left(q\right)+{\mathrm{SSNR}}_{\mathrm{tcDF}}\left(q\right)},\\ w_{\mathrm{tcDF}}\left(q\right) & = & 1-w_{\mathrm{ptycho}}\left(q\right). \end{array}$$

### 4.10 FF‐STEM Reconstruction

Let ${\hat{O}}_{\mathrm{ptycho}}\left(q\right)$ and ${\hat{O}}_{\mathrm{tcDF}}\left(q\right)$ denote the Fourier transforms of the ptychographic and tcDF reconstructions. The fused spectrum is

(40) $${\hat{I}}_{\mathrm{FF}-\mathrm{STEM}}\left(q\right)=w_{\mathrm{ptycho}}\left(q\right)\;\hat{O}\left(q\right)+w_{\mathrm{tcDF}}\left(q\right)\;{\hat{O}}_{\mathrm{tcDF}}\left(q\right),$$

and the FF‐STEM reconstruction is obtained by the inverse Fourier transform

(41) $$I_{\mathrm{FF}-\mathrm{STEM}}\left(r\right)=F^{-1}\;\left[{\hat{I}}_{\mathrm{FF}}\left(q\right)\right]\left(r\right).$$

Because the noise contributions of the two channels are independent (see below), the noise variance of the fused image combines inverse‐additively at each spatial frequency,

(42) $$\frac{1}{\sigma_{\text{FF-STEM}}^{2}\left(q\right)}=\frac{1}{\sigma_{\mathrm{ptycho}}^{2}\left(q\right)}+\frac{1}{\sigma_{\mathrm{tcDF}}^{2}\left(q\right)},$$

which is the smallest noise variance achievable by any band‐passthrough linear combination of the two channels. When the per‐channel SSNRs are reported against a common reference signal‐power spectrum (e.g., the band‐blended composite used to estimate the half‐data SSNR for tcDF), this inverse‐noise‐variance addition corresponds to addition of the SSNRs,

(43) $${\mathrm{SSNR}}_{\text{FF-STEM}}\left(q\right)\approx {\mathrm{SSNR}}_{\mathrm{ptycho}}\left(q\right)+{\mathrm{SSNR}}_{\mathrm{tcDF}}\left(q\right),$$

which we report in Figure 1e as a summary diagnostic of the combined frequency‐domain information content of the two channels.

### 4.11 Limits of the Fused‐Image Interpretation

Two consequences follow directly from $S_{1}\left(q\right)\neq S_{2}\left(q\right)$ in general:

(a) *No single‐functional interpretation*. The fused image cannot be interpreted, frequency by frequency, as a quantitative reconstruction of any single physical functional of the specimen. At low spatial frequencies it is dominated by the tcDF (incoherent amplitude) channel; at high spatial frequencies it is dominated by the direct‐ptychography (coherent phase) channel [19]. The transition is governed by the SSNR ratio, not by a perturbative assumption.
(b) *Crossover‐band caveat*. In the narrow band where both channels contribute, the fused image is a band‐blended composite whose precise quantitative interpretation is not analytically known and depends on the specimen.

The path to a quantitative recovery of the projected potential, beyond a high‐contrast composite image, is iterative multislice ptychography for the full‐field dataset: a strong‐phase complex transmission model handles the σV phase term, the $\sigma^{2}V^{2}$ coherent amplitude term, and multiple elastic scattering explicitly within its forward model. The incoherent amplitude contribution to the dark‐field signal (Eq. 33 of [19]) is only captured implicitly by such a partially coherent forward model and leads to a systematic underestimation of the sample extent in the depth direction [45] and the peak amplitude of the electrostatic potential [46]; quantitative recovery of the dark‐field channel requires a Bloch‐wave treatment of the elastic dark‐field scattering (Sec. 3.5 of [19]) or a mixed‐object‐state [47] / frozen‐phonon extension [48], both of which come with a substantial increase in computational costs.

Every reconstruction method in 4D‐STEM rests on a specific set of approximations and inherits a corresponding catalogue of systematic errors. The weak‐phase approximation in direct ptychography, assumption of defocus‐only aberrations tcDF, the coherent forward model and static‐potential / no‐inelastic assumption in standard iterative multislice ptychography, the depth‐extent underestimation noted above for thicker specimens, and so on. Awareness of these approximations, and of the resulting systematic errors in the recovered image or potential, is essential for any quantitative interpretation. At the same time, the most accurate forward model that one can presently write, a multislice formulation augmented by a mixed‐object‐state and frozen‐phonon ensemble and a low‐rank probe density matrix, applied iteratively, is computationally expensive, parameter‐heavy, and in most practical cases unnecessary to answer the materials‐science or structural‐biology question at hand. The choice of method should therefore be guided by the question being asked: when local structure, interface chemistry, particle morphology, or biological context is the target, a fast method whose approximations are explicit and whose systematic errors are bounded is often preferable to a more accurate method that cannot be run on the available data within a reasonable time. FF‐STEM is presented in this spirit, as a fast, dose‐efficient, contrast gap‐free composite imaging method whose approximations and limits of validity are made explicit (Sec. “Limits of the linear fusion” in the Discussion), and which is intended to complement iterative reconstructions when the latter are warranted. We use the raw tcDF reconstruction as the default input to the fusion; tests with square‐root‐transformed tcDF show that such nonlinear compression can improve contrast balance at high SNR but is not generally advantageous at low dose, where it amplifies weak‐signal background fluctuations (Figure S7).

### 4.12 Noise Independence of the Ptychographic and tcDF Channels

The Wiener band composite in Equation (38) requires only that the *noise* contributions of the two channels are uncorrelated — not that they encode the same noise‐free signal. This noise independence is well justified for FF‐STEM.

The two channels are formed from disjoint subsets of the diffraction data. The ptychographic reconstruction uses only the bright‐field disc, whereas the tcDF reconstruction is computed from azimuthal segments in the dark‐field ring. At the detector level, the dominant noise source is shot noise, which is uncorrelated between different pixels. Since the bright‐field and dark‐field regions are non‐overlapping, the corresponding noise realizations entering the ptychographic and tcDF pipelines are independent. The reconstruction algorithms are furthermore linear with respect to the measured intensities (although the underlying contrast mechanisms are not, see [19]): direct ptychography forms a complex aperture‐overlap‐weighted sum of the BF data, and tcDF reconstructs tilt‐corrected dark‐field images from angle‐resolved intensities by linear shift‐and‐sum operations. Noise from independent detector pixels therefore propagates linearly through both pipelines and remains uncorrelated between channels. We therefore model the ptychographic and tcDF Fourier coefficients as

(44) $$\begin{array}{ccc} {\hat{O}}_{\mathrm{ptycho}}\left(q\right) & = & S_{\mathrm{ptycho}}\left(q\right)+n_{\mathrm{ptycho}}\left(q\right),\\ {\hat{O}}_{\mathrm{tcDF}}\left(q\right) & = & S_{\mathrm{tcDF}}\left(q\right)+n_{\mathrm{tcDF}}\left(q\right), \end{array}$$

with

(45) $$\begin{array}{ccc} E\;n_{\mathrm{ptycho}} & = & E\;n_{\mathrm{tcDF}}=0,\;\mathrm{Cov}\\ \left[n_{\mathrm{ptycho}}\left(q\right),n_{\mathrm{tcDF}}\left(q^{\prime }\right)\right] & = & 0\;\forall \;q,q^{\prime }. \end{array}$$

The noise‐free signals $S_{\mathrm{ptycho}}\left(q\right)$ and $S_{\mathrm{tcDF}}\left(q\right)$ are distinct contrast functionals of the projected potential and are not assumed to be equal. Under this model, the SSNR‐based fusion in Equation (41) is the minimum‐noise‐variance Wiener band composite of the two channels under the band‐passthrough constraint, with the interpretation given above.

### 4.13 Relation to Recent Contrast Theory

Following the contrast decomposition of [19] (their Eq. 7 and Sec. 3.4), the bright‐field disk simultaneously carries (i) a coherent phase term proportional to σV (Friedel‐symmetric, odd in defocus), (ii) a coherent amplitude term proportional to $\sigma^{2}V^{2}$ (anti‐Friedel, even in defocus), while the dark‐field region carries (iii) an incoherent amplitude term, also of order $\sigma^{2}V^{2}$, but with a distinct functional dependence on V (their Eq. 33). Direct ptychography, as we use it here, projects the BF data onto the sinχ Friedel channel and is therefore primarily sensitive to (i); tcDF synthesizes (iii) from the dark‐field region. The two channels are therefore intentionally complementary contrast functionals.

Two consequences are worth noting. First, by symmetry of the ptychographic reconstruction filter, the $\sigma^{2}V^{2}$ coherent‐amplitude term in the BF region is suppressed at the frequencies where the ptychographic channel actually contributes to the fused image; conversely, the band‐disjoint structure of the Wiener weights ($w_{\mathrm{ptycho}}\to 0$ at low spatial frequencies, $w_{\mathrm{tcDF}}\to 0$ at high spatial frequencies) ensures that the residual mixing of the two $\sigma^{2}V^{2}$ terms is small in practice. Second, the different Z‐dependence of the phase and amplitude / dark‐field channels [19] therefore manifests in the fused image at low frequencies as enhanced mass‐thickness contrast, the desired behavior for the band that direct ptychography cannot reach, while atomic‐resolution Z‐ratios at high frequencies track those of the σV phase channel, as quantified by the Co/O atomic‐column ratios reported in Figure 2.

An immediate variant suggested by this analysis is to replace the WPOA‐based direct‐ptychography BF channel used here with a BF method that does not drop the $\sigma^{2}V^{2}$ coherent‐amplitude term. *Wigner‐distribution deconvolution* (WDD) [16, 17] without the WPOA assumption, which can in principle recover the full complex object exp(iσV) within the first‐Born approximation and therefore captures both coherent terms beyond the perturbative regime. This would close the perturbation‐order self‐consistency by construction at a different point in the cost / generality trade‐off. Previous reports [49, 50] compare the weak‐phase single‐sideband reconstruction and WDD and from those reconstructions it can be seen that the overall overall contrast difference between the weak‐phase and strong‐phase reconstruction is negligible, while the strong‐phase reconstruction involves an addition FFT of the whole dataset, therefore we have opted for the faster method in this work since the goal is fast feedback. The Wiener band‐composite framework presented here applies unchanged: the fusion is determined by the per‐channel SSNRs, and the choice of BF method enters only through the per‐channel SSNR curves.

### 4.14 Limits of the Linear Fusion

From a theoretical standpoint, the FF‐STEM fusion framework as presented here makes no claim to recover a single quantitative functional of the projected potential. The fused image is a Wiener band composite of two channels with distinct contrast functionals; its quantitative interpretation is therefore band‐dependent (low frequencies: incoherent amplitude / mass‐thickness; high frequencies: coherent phase). For thin, weakly scattering specimens this composite is high‐contrast, dose‐efficient, and faithful to the band‐by‐band physics described above. For strongly scattering or thick specimens, however, the leading‐order contrast description breaks down: dynamical diffraction, channeling, thickness‐dependent scattering, and the breakdown of the first Born approximation make the channel signals nonlinear in V, and partial coherence and inelastic scattering further damp and distort each channel's transfer.

That said, our simulations allow a direct quantitative comparison with the ground truth potential and show that practically, FF‐STEM achieves a quantitative improvement in SSIM of 28% compared to a state‐of‐the‐art near‐real‐time BF‐only method for the carbon wedge sample in Figure 2. Similarly for a sample spanning 5 rows of the periodic table in Figure S7, FF‐STEM delivers a 34% SSIM improvement (0.585 to 0.786) for low‐dose and high‐dose datasets when compared to direct ptychography. No universal thickness threshold can be assigned, because the onset of nonlinear effects depends on composition, crystallographic orientation, accelerating voltage, convergence angle, detector geometry, and the spatial‐frequency band of interest. Figure S4 shows that FF‐STEM degrades more gracefully than direct ptychography, not losing lattice visibility even for a 40nm thick ${\mathrm{SrTiO}}_{3}$ wedge.

For thick light‐element biological specimens at 300 kV, the weak‐scattering condition is not satisfied for the full projected specimen thickness. The mitochondrion dataset should therefore not be interpreted as a quantitative reconstruction of V, but as a demonstration of dose‐efficient high‐contrast biological imaging. This interpretation is consistent with the original tcBF‐STEM study, which demonstrated improved contrast and dose efficiency for thick biological samples beyond 500 nm while noting that multiple elastic scattering increasingly limits the coherent phase‐contrast signal as the thickness approaches the elastic mean free path [22]. While multi‐slice ptychography theoretically models the different contrast terms more accurately, to date no successful multi‐slice ptychography reconstruction of thick biological specimens beyond 500 nm has been presented.

### 4.15 Inelastic Scattering

In a thick specimen, the majority of electrons experience both elastic and inelastic scattering. However, elastic contrast remains largely intact even when electrons are scattered into inelastic channels [28, 51]. This can be attributed to the fact that the most probable inelastic scattering events are significantly more delocalized than elastic ones. As a result, they introduce only weak, low‐frequency modulations in real space and cause minimal broadening of the angular distribution [22, 52].

### 4.16 Partial Coherence

The ptychographic SSNR (Equation (13)) is derived under the assumption of a fully coherent probe. In practice, partial spatial and temporal coherence introduce additional envelope functions that damp the transfer at high spatial frequencies [30, 53]. The tcDF channel, being predominantly incoherent (amplitude contrast), is relatively insensitive to the coherence envelope. The direct ptychography channel could be adapted to include the temporal and spatial envelope functions, as done in [30]. To this end, the source size and chromatic defocus blur would need to be determined for the microscope. Our current implementation does not include this feature to minimize the reconstruction time.

### 4.17 EF‐TEM Experimental Parameters

The data from the published EFTEM dataset [22] were recorded at 300kV with a Falcon 4i detector with a Selectrix X energy filter.

### 4.18 4D‐STEM Experimental Parameters

The experiments with the TimePix4 detector were performed on a TFS Spectra (S)TEM microscope equipped with X‐CFEG and a probe corrector, operated at 60kV. The 4D‐STEM datasets were recorded at a beam current of 36 pA and a dwell time of 1 microsecond.

The experiments using the ARINA detector were performed on a probe‐corrected TFS Spectra 30‐200 microscope equipped with XCFEG.

The experiments using the 4DCamera were performed on the TEAM 0.5 microscope at the National Center for Electron Microscopy, Berkeley, a double‐corrected TFS Titan microscope. All experimental parameters are listed in Table 1.

**TABLE 1:** Experimental parameters corresponding to Figures 1, 2, 3, 4, 5, 6.

Figure	Sample	Energy (keV)	α (mrad)	Fluence (e/Å 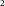 )	Scan step (Å)	Det. pixel (Å 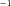 )	Det. Nyquist (Å 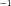 )	Defocus (Å)
Figure 1	${\mathrm{Gd}}_{2}$ $O_{3}$	60	30	$1.2\times 10^{3}$	0.61	0.034	1.904	44
Figure 2a–d	${\mathrm{Co}}_{3}$ $O_{4}$	200	21	$1.0\times 10^{3}$	0.20	0.078	2.496	200
Figure 2h–k	Amorph. C	300	19.68	$28.5\times 10^{3}$	0.25	0.042	2.016	200
Figure 3	CNT/${\mathrm{TaTe}}_{2}$	80	25	$30.5\times 10^{3}$	0.316	0.029	1.595	278
Figure 4a	Diffr. grating	200	30	$3.4\times 10^{3}$	0.727	0.052	2.496	160
Figure 4b	${\mathrm{Gd}}_{2}$ $O_{3}$	60	30	$1.2\times 10^{3}$	0.43	0.034	1.904	83
Figure 4c	CNT/${\mathrm{TaTe}}_{2}$	80	25	$30.5\times 10^{3}$	0.25	0.042	1.595	278
Figure 5	Virus‐like particle	200	30.6	32	11	0.018	0.765	140
Figure 6	Mitochondrion	300	7.0	14	28.7	0.007	0.4375	21815

### 4.19 Viral‐Like Particle Preparation and Grid Preparation

Virus‐like particles used in the experiment represent immature equine infectious anemia virus (EIAV) that, under chosen in vitro conditions, assemble into a mixture of spheres and tubes. The viral sample was prepared according to the protocol described in ref. [54].

The CA–NC domains of EIAV Gag were cloned into a pET28 vector in ORF with an N‐terminal ${\mathrm{His}}_{6}$–SUMO tag using a standard restriction–ligation molecular cloning method. The protein was expressed in *E. coli* BL21(DE3) cells. All purification steps were carried out at $4^{\circ }C$ or on ice. Cell pellets were resuspended in buffer (20 mM Tris–HCl pH 8, 500 mM NaCl, 2 mM TCEP, 5 μM ${\mathrm{ZnCl}}_{2}$), disrupted by sonication, and clarified by centrifugation. Nucleic acid was removed by adding 0.03% (v/v) polyethyleneimine, and the resulting precipitate was removed by centrifugation. The target protein was then enriched by precipitation with 20% ammonium sulfate, and the pellet was resuspended in the same buffer as used for affinity chromatography (20 mM Tris–HCl pH 8, 500 mM NaCl, 2 mM TCEP). The resuspended material was filtered (0.2 μm) and loaded onto a ${\mathrm{Ni}}^{2+}$‐affinity column, followed by elution with imidazole. The eluate was dialyzed overnight in the presence of ULP1 protease and was reapplied on a second ${\mathrm{Ni}}^{2+}$ column to remove the cleaved SUMO tag and the ULP1 protease. The final purified protein was flash‐frozen in liquid nitrogen and stored at $-80^{\circ }C$ in storage buffer (20 mM Tris–HCl pH 8, 500 mM NaCl, 2 mM TCEP).

The assembly reaction was carried out in a buffer consisting of 50 mM MES (pH 6.0), 100 mM NaCl, 2 mM TCEP, supplemented with 10 μM IP6 and 10 μM GT50. Reactions were incubated overnight at $4^{\circ }C$ and remained stable for several weeks thereafter. Grids were prepared on the Leica GP2 using backside blotting under 90% humidity at $10^{\circ }C$. Before vitrification, the sample was mixed with 10 nm gold fiducial beads at a 1:10 (beads:sample) ratio. For grid preparation, 2 μL of the mixture was applied to the reverse side of the grid, and an additional 2.5 μL was applied to the front. Blotting was performed from the back for 3.5 s.

### 4.20 Runtime Performance

**FIGURE 7:**
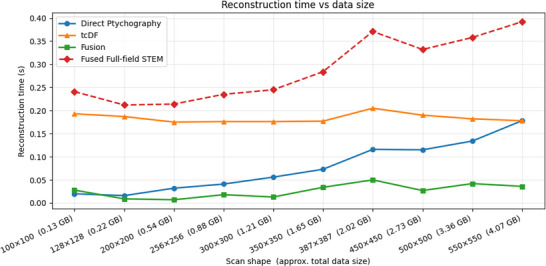
Reconstruction time as a function of 4D‐STEM data size. Measured reconstruction time for direct ptychography (blue), tcDF (orange), FF‐STEM (green), and the total combined time (red). Each dataset corresponds to a cropped scan region of the full 4D‐STEM acquisition. The approximate total data size (assuming 32‐bit float) is indicated below the horizontal axis.

We evaluate the runtime performance of our FF‐STEM implementation by varying the number of scan positions used from the same experimental 4D‐STEM dataset. As shown in Figure 7, both the direct‐ptychography and tcDF reconstructions exhibit nearly constant execution times across different scan sizes, reflecting their analytical, non‐iterative formulation. The total runtime scales linearly with data volume, remaining below 0.5s even for the 550 × 550 × 85 × 85 dataset. This performance demonstrates the high computational efficiency of our CUDA‐accelerated implementation and confirms that near‐real‐time reconstruction and fusion of megapixel‐scale 4D‐STEM data are feasible on a modern graphics processing unit. All benchmarks were performed on a single NVIDIA H200 NVL (144 GB) using custom CUDA kernels integrated into a Python/PyTorch framework. Once the direct ptychography and tcDF reconstructions are computed, the reconstructed images are stored in GPU memory, and the fusion step is performed by extracting the reconstructed images on the GPU without data transfer overhead. The total runtime (red line in Figure 7) measurements include the full pipeline from raw 4D‐STEM data to the final FF‐STEM image, encompassing all reconstruction and fusion steps. We emphasize that these timings represent the absolute runtime of the FF‐STEM pipeline, not a comparison with external reconstruction methods. The combination of accuracy, multi‐frequency contrast, and sub‐second runtime positions the FF‐STEM method as a practical framework for live imaging and feedback during experiments. Due to its linear operation, it is compatible with live reconstructions from multi‐segment detectors or streamed data from pixelated detectors, like OBF‐STEM [15] and various live ptychography implementations [55, 56].

### 4.21 Quantitative Image‐Quality Metrics

The following *contrast metrics* (single‐image, no reference required) are used:

#### 4.21.1 Contrast (line profiles)

For line‐profile comparisons we use the Weber contrast,

(46) $$C_{\mathrm{Weber}}=\left(I_{\max}-I_{\min}\right)/I_{\mathrm{mean}},$$

a single‐image metric that quantifies signal modulation along a line profile relative to its local mean intensity.

#### 4.21.2 Atomic‐column intensity

For atomic‐column comparisons in the ${\mathrm{Co}}_{3}O_{4}$ simulation, we fit a local two‐dimensional Gaussian with a constant background to each selected column,

(47) $$I\left(x,y\right)=B+A\exp[-\left({\left(x-x_{0}\right)}^{2}+{\left(y-y_{0}\right)}^{2}\right)/\left(2\sigma^{2}\right)].$$

The fitted amplitude A is used to quantify the atomic‐column intensity above the local background B, and the latter to quantify the local background level. Column‐intensity ratios and uncertainties are reported from multiple fitted Co and O columns.

### 4.22 Optimal Defocus for FF‐STEM

For practical experiments, the scan step and defocus should be chosen together. The scan step is first constrained by the desired field of view, dose budget, probe overlap, and target spatial resolution. For defocused 4D‐STEM reconstructions, it also determines how much defocus is needed to generate sufficient parallax diversity: a coarser scan step generally requires a larger defocus‐induced virtual‐image shift [41], whereas a finer scan step allows a smaller defocus. In FF‐STEM, this choice is a compromise between the two fused channels. Finite defocus benefits the direct‐ptychography channel by improving low‐ and medium‐spatial‐frequency transfer and by enabling scan upsampling, but the tcDF channel is affected differently: it provides robust low‐frequency dark‐field contrast, while excessive defocus can broaden the tilt‐corrected dark‐field reconstruction and reduce its useful mid‐ and high‐frequency content. Therefore, the preferred acquisition condition is not the optimum of either direct ptychography or tcDF alone, but a moderate defocus that provides sufficient parallax diversity relative to the scan step while keeping the tcDF image sufficiently sharp. Because FF‐STEM can be reconstructed nearly in real time, this choice can be refined experimentally: an initial 4D‐STEM dataset can be reconstructed immediately, the direct‐ptychography, tcDF, and fused images inspected, and the corresponding SSNR curves used to adjust the defocus and scan step toward the spatial‐frequency range most relevant for the sample. This practical tuning procedure is illustrated in Figure S2, where changing the probe defocus modifies the FF‐STEM SSNR near the low‐frequency crossover while preserving the recovery of mesoscale real‐space features. We provide an interactive widget for optimization of SSNR given experimental settings at this URL.

## Author Contributions

S.Y., P.P. and G.V. implemented FF‐STEM algorithms. S.Y. performed experimental reconstructions and prepared the initial manuscript. G.V. and P.P. derived and discussed SSNR and fusion weights. S.Y., M.W and E.S performed diffraction grating experiments. D.S. and P.P. performed cryo‐4D‐STEM experiment with VLPs. N.P. and S.S. performed 4D‐STEM simulations and reconstructions of simulated data. S.K., R.E., S.V. performed Timepix4 experiments with $Gd_{2}O_{3}$. F.S. and D.C. prepared frozen‐hydrated virus‐like particles and trained P.P in cryo‐EM. B.Z. and X.Y. prepared $Gd_{2}O_{3}$ nanohelices. P.P. conceived the study. All authors read and approved the final version of the manuscript.

## Funding

This project has received funding from the European Research Council (ERC) under the European Union's Horizon 2020 research and innovation programme (Project HyperScaleEM, Grant agreement No. 101164581) and from the Deutsche Forschungsgemeinschaft (DFG, German Research Foundation) through the Research Training Group GRK 3103 CorMic: Korrelative Materialmikroskopie – Von nanostrukturierten funktionalen Filmen zu hierarchischen Funktionsmaterialien (project number 537140136). B.Z. and X.Y. were supported by the U.S. National Science Foundation under award CHE‐2404338. X.Y. also thanks the Principal Investigator Development in Sustainability Grant from the American Chemical Society.

## Conflicts of Interest

The authors declare no conflicts of interest.

## Availability of Data and Materials

The data that support the findings of this study are openly available in Zenodo at https://doi.org/10.5281/zenodo.18008901. The reconstruction code is available as an open‐source repository at the scatterem github repo.

## Supporting information


**Supporting File**: advs76620‐sup‐0001‐SuppMat.pdf.

## References

[1] B. D. Levin , “Direct Detectors and Their Applications in Electron Microscopy for Materials Science,” Journal of Physics: Materials 4, no. 4 (2021): 042005.

[2] M. W. Tate , P. Purohit , D. Chamberlain , et al., “High Dynamic Range Pixel Array Detector for Scanning Transmission Electron Microscopy,” Microscopy and Microanalysis 22, no. 1 (2016): 237–249. 26750260 10.1017/S1431927615015664

[3] C. Ophus , “Four‐Dimensional Scanning Transmission Electron Microscopy (4D‐STEM): From Scanning Nanodiffraction to Ptychography and Beyond,” Microscopy and Microanalysis 25, no. 3 (2019): 563–582. 31084643 10.1017/S1431927619000497

[4] H. T. Philipp , M. W. Tate , K. S. Shanks , et al., “Very‐High Dynamic Range, 10,000 Frames/Second Pixel Array Detector for Electron Microscopy,” Microscopy and Microanalysis 28, no. 2 (2022): 425–440. 10.1017/S1431927622000174 35249574

[5] A. M. Maiden and J. M. Rodenburg , “An Improved Ptychographical Phase Retrieval Algorithm for Diffractive Imaging,” Ultramicroscopy 109, no. 10 (2009): 1256–1262. 19541420 10.1016/j.ultramic.2009.05.012

[6] Y. Jiang , Z. Chen , Y. Han , et al., “Electron Ptychography of 2D Materials to Deep Sub‐ångström Resolution,” Nature 559, no. 7714 (2018): 343–349. 30022131 10.1038/s41586-018-0298-5

[7] Z. Chen , Y. Jiang , Y.‐T. Shao , et al., “Electron Ptychography Achieves Atomic‐Resolution Limits Set by Lattice Vibrations,” Science 372, no. 6544 (2021): 826–831. 34016774 10.1126/science.abg2533

[8] P. M. Pelz , S. M. Griffin , S. Stonemeyer , et al., “Solving Complex Nanostructures with Ptychographic Atomic Electron Tomography,” Nature Communications 14, no. 1 (2023): 7906. 10.1038/s41467-023-43634-z PMC10689721 38036516

[9] A. Romanov , M. G. Cho , M. C. Scott , and P. Pelz , “Multi‐Slice Electron Ptychographic Tomography for Three‐Dimensional Phase‐Contrast Microscopy Beyond the Depth of Focus Limits,” Journal of Physics: Materials 8, no. 1 (2024): 015005.

[10] S. You , A. Romanov , and P. M. Pelz , “Near‐Isotropic Sub‐ångstrom 3D Resolution Phase‐Contrast Imaging Achieved by End‐to‐End Ptychographic Electron Tomography,” Physica Scripta 100, no. 1 (2024): 015404.

[11] K. Müller‐Caspary , F. F. Krause , T. Grieb , et al., “Measurement of Atomic Electric Fields and Charge Densities from Average Momentum Transfers Using Scanning Transmission Electron Microscopy,” Ultramicroscopy 178 (2017): 62–80. 27217350 10.1016/j.ultramic.2016.05.004

[12] R. Close , Z. Chen , N. Shibata , and S. Findlay , “Towards Quantitative, Atomic‐Resolution Reconstruction of the Electrostatic Potential via Differential Phase Contrast Using Electrons,” Ultramicroscopy 159 (2015): 124–137. 26381331 10.1016/j.ultramic.2015.09.002

[13] I. Lazić , E. G. Bosch , and S. Lazar , “Phase Contrast STEM for Thin Samples: Integrated Differential Phase Contrast,” Ultramicroscopy 160 (2016): 265–280. 26590505 10.1016/j.ultramic.2015.10.011

[14] K. Müller‐Caspary , F. F. Krause , F. Winkler , et al., “Comparison of First Moment STEM with Conventional Differential Phase Contrast and the Dependence on Electron Dose,” Ultramicroscopy 203 (2019): 95–104. 30660404 10.1016/j.ultramic.2018.12.018

[15] K. Ooe , T. Seki , M. Nogami , Y. Ikuhara , and N. Shibata , “Dose‐Efficient Phase‐Contrast Imaging of Thick Weak Phase Objects via OBF STEM Using a Pixelated Detector,” Microscopy 74, no. 2 (2025): 98–106. 39506558 10.1093/jmicro/dfae051 PMC11957251

[16] J. Rodenburg and R. Bates , “The Theory of Super‐Resolution Electron Microscopy via Wigner‐Distribution Deconvolution,” Philosophical Transactions of the Royal Society of London: Series A: Physical and Engineering Sciences 339, no. 1655 (1992): 521–553.

[17] T. J. Pennycook , A. R. Lupini , H. Yang , M. F. Murfitt , L. Jones , and P. D. Nellist , “Efficient Phase Contrast Imaging in STEM Using a Pixelated Detector. Part 1: Experimental Demonstration at Atomic Resolution,” Ultramicroscopy 151 (2015): 160–167. 25458189 10.1016/j.ultramic.2014.09.013

[18] H. Yang , R. Rutte , L. Jones , et al., “Simultaneous Atomic‐Resolution Electron Ptychography and Z‐Contrast Imaging of Light and Heavy Elements in Complex Nanostructures,” Nature Communications 7, no. 1 (2016): 12532. 10.1038/ncomms12532 PMC5007440 27561914

[19] D. Ma , G. Li , D. A. Muller , and S. E. Zeltmann , “Information in 4D‐STEM: Where It Is, and How to Use It,” Ultramicroscopy 283 (2026): 114351. 41880920 10.1016/j.ultramic.2026.114351

[20] K. X. Nguyen , P. Purohit , R. Hovden , et al., “4D‐STEM for Quantitative Imaging of Magnetic Materials with Enhanced Contrast and Resolution,” Microscopy and Microanalysis 22, no. S3 (2016): 1718–1719.

[21] K. A. Spoth , K. X. Nguyen , D. A. Muller , and L. F. Kourkoutis , “Dose‐Efficient Cryo‐STEM Imaging of Whole Cells Using the Electron Microscope Pixel Array Detector,” Microscopy and Microanalysis 23, no. S1 (2017): 804–805.

[22] Y. Yu , K. A. Spoth , M. Colletta , et al., “Dose‐Efficient Cryo‐Electron Microscopy for Thick Samples Using Tilt‐Corrected Scanning Transmission Electron Microscopy,” Nature Methods 22.10 (2025): 2138–2148. 40987871 10.1038/s41592-025-02834-9 PMC12510875

[23] G. Varnavides , S. M. Ribet , S. E. Zeltmann , et al., “Iterative Phase Retrieval Algorithms for Scanning Transmission Electron Microscopy,” arXiv Preprint arXiv:2309.05250 (2023).

[24] D. Ma , D. A. Muller , and S. E. Zeltmann , “Using Aberrations to Improve Dose‐Efficient Tilt‐Corrected 4D‐STEM Imaging,” Microscopy and Microanalysis 32, no. 2 (2026): ozag008. 42054153 10.1093/mam/ozag008

[25] P. Hartel , H. Rose , and C. Dinges , “Conditions and Reasons for Incoherent Imaging in STEM,” Ultramicroscopy 63, no. 2 (1996): 93–114.

[26] P. Nellist and S. Pennycook , “The Principles and Interpretation of Annular Dark‐Field Z‐Contrast Imaging,” in Advances in Imaging and Electron Physics, vol. 113 (Elsevier, 2000), 147–203.

[27] H. Yang , R. Rutte , L. Jones , et al., “Simultaneous Atomic‐Resolution Electron Ptychography and Z‐Contrast Imaging of Light and Heavy Elements in Complex Nanostructures,” Nature Communications 7 (2016): 12532. 10.1038/ncomms12532 PMC5007440 27561914

[28] H. Rose , “Nonstandard Imaging Methods in Electron Microscopy,” 2 (January 1976): 251–267. 10.1016/s0304-3991(76)91538-2 888244

[29] G. Varnavides , J. M. Bekkevold , S. M. Ribet , M. C. Scott , L. Jones , and C. Ophus , “Beyond Contrast Transfer: Spectral SNR as a Dose‐Aware Metric for STEM Phase Retrieval,” arXiv:2507.19476 (2025). 10.1093/mam/ozag005 42054152

[30] F. Bennemann , A. I. Kirkland , D. A. Muller , and P. Nellist , “Detective Quantum Efficiency Based Comparison of HRTEM and Ptychography Phase Imaging,” arXiv:2509.12037 (2025), [cond‐mat]. 10.1093/mam/ozag018 42054150

[31] M. Unser , B. L. Trus , and A. C. Steven , “A New Resolution Criterion Based on Spectral Signal‐to‐Noise Ratios,” Ultramicroscopy 23, no. 1 (Jan. 1987): 39–51. 3660491 10.1016/0304-3991(87)90225-7

[32] M. V. Heel and M. Schatz , “Fourier Shell Correlation Threshold Criteria Q,” Journal of Structural Biology 151 (2005): 250–262, citation Key: Heel2005 00000 . 16125414 10.1016/j.jsb.2005.05.009

[33] Z. Wang , A. C. Bovik , H. R. Sheikh , and E. P. Simoncelli , “Image Quality Assessment: From Error Visibility to Structural Similarity,” IEEE Transactions on Image Processing 13, no. 4 (2004): 600–612. 15376593 10.1109/tip.2003.819861

[34] P. M. Pelz , H. G. Brown , S. Stonemeyer , et al., “Phase‐Contrast Imaging of Multiply‐Scattering Extended Objects at Atomic Resolution by Reconstruction of the Scattering Matrix,” Physical Review Research 3, no. 2 (2021): 023159.

[35] Y. Liu , Y. Li , S. Jeong , Y. Wang , J. Chen , and X. Ye , “Colloidal Synthesis of Nanohelices via Bilayer Lattice Misfit,” Journal of the American Chemical Society 142, no. 29 (2020): 12777–12783. 32559376 10.1021/jacs.0c05175

[36] C. O'Leary , C. Allen , C. Huang , et al., “Phase Reconstruction Using Fast Binary 4D STEM Data,” Applied Physics Letters 116, no. 12 (2020): 124101.

[37] B. Yuan , Z. Wang , S. Zhang , et al., “Atomically Resolved Edges and Defects in Lead Halide Perovskites,” Nature 647.8089 (2025): 364–368. 41162710 10.1038/s41586-025-09693-6

[38] C. Huang , J. S. Kim , and A. I. Kirkland , “Cryo‐Electron Ptychography: Applications and Potential in Biological Characterisation,” Current Opinion in Structural Biology 83 (2023): 102730. 37992450 10.1016/j.sbi.2023.102730

[39] S. Seifer , L. Houben , and M. Elbaum , “Shadow Montage and Cone‐Beam Reconstruction in 4D‐STEM Tomography,” p. 2025.09.06.674643, 2025. 10.1093/mam/ozaf126 41533762

[40] H. Yang , P. Ercius , P. D. Nellist , and C. Ophus , “Enhanced Phase Contrast Transfer Using Ptychography Combined with a Pre‐Specimen Phase Plate in a Scanning Transmission Electron Microscope,” Ultramicroscopy 171 (Dec. 2016): 117–125. 00013. 27664566 10.1016/j.ultramic.2016.09.002

[41] G. Varnavides , J. M. Bekkevold , S. M. Ribet , M. C. Scott , L. Jones , and C. Ophus , “Relaxing Direct Ptychography Sampling Requirements via Parallax Imaging Insights,” Microscopy and Microanalysis 32, no. 2 (2026): ozaf139. 42054151 10.1093/mam/ozaf139

[42] H. Rose , “Phase Contrast in Scanning Transmission Electron Microscopy,” Optik 39, no. 4 (1974): 416–436.

[43] L. Loetgering , M. Du , K. S. Eikema , and S. Witte , “ZPIE: An Autofocusing Algorithm for Ptychography,” Optics Letters 45, no. 7 (2020): 2030–2033. 32236060 10.1364/OL.389492

[44] O. L. Krivanek , N. Dellby , and A. R. Lupini , “Towards Sub‐Å Electron Beams,” Ultramicroscopy 78, no. 1–4 (1999): 1–11. 00457.

[45] H. KP , X. Wei , C.‐H. Lee , et al., “Mind the Gap—Imaging Buried Interfaces in Twisted Oxide Moires,” Advanced Materials 38, no. 18 (2026): e21189. 41766510 10.1002/adma.202521189

[46] Z. Herdegen , B. Diederichs , and K. Müller‐Caspary , “Thermal Vibrations in the Inversion of Dynamical Electron Scattering,” Physical Review B 110 (Aug. 2024): 064102.

[47] A. Gladyshev , B. Haas , T. C. Pekin , et al., “Electron Ptychography Reveals Correlated Lattice Vibrations at Atomic Resolution,” arXiv Preprint arXiv:2309.12017 (2025). 10.1038/s41467-026-74135-4 PMC13254316 42270641

[48] B. Diederichs , Z. Herdegen , A. Strauch , F. Filbir , and K. Muller‐Caspary , “Exact Inversion of Partially Coherent Dynamical Electron Scattering for Picometric Structure Retrieval,” Nature Communications 15 (2024): 101. 10.1038/s41467-023-44268-x PMC10762228 38168078

[49] H. Yang , I. MacLaren , L. Jones , et al., “Electron Ptychographic Phase Imaging of Light Elements in Crystalline Materials Using Wigner Distribution Deconvolution,” Ultramicroscopy 180 (2017): 173–179. 28434783 10.1016/j.ultramic.2017.02.006

[50] C. M. O'Leary , G. T. Martinez , E. Liberti , M. J. Humphry , A. I. Kirkland , and P. D. Nellist , “Contrast Transfer and Noise Considerations in Focused‐Probe Electron Ptychography,” Ultramicroscopy 221 (2021): 113189. 33360480 10.1016/j.ultramic.2020.113189

[51] S. Cundy , A. Howie , and U. Valdrè , “Preservation of Electron Microscope Image Contrast After Inelastic Scattering,” Philosophical Magazine 20, no. 163 (1969): 147–163.

[52] R. F. Egerton , Electron Energy‐Loss Spectroscopy in the Electron Microscope (Springer Science & Business Media, 2011).

[53] P. Nellist and J. Rodenburg , “Beyond the Conventional Information Limit: The Relevant Coherence Function,” Ultramicroscopy 54, no. 1 (1994): 61–74.

[54] R. A. Dick , C. Xu , D. R. Morado , et al., “Structures of Immature EIAV Gag Lattices Reveal a Conserved Role for IP6 in Lentivirus Assembly,” PLOS Pathogens 16, no. 1 (2020): e1008277. 31986188 10.1371/journal.ppat.1008277 PMC7004409

[55] H. L. Lalandec Robert , M. Leo Leidl , K. Müller‐Caspary , and J. Verbeeck , “Benchmarking Analytical Electron Ptychography Methods for the Low‐Dose Imaging of Beam‐Sensitive Materials,” European Physical Journal: Applied Physics 100 (2025): 1–37.

[56] A. Bangun , P. F. Baumeister , A. Clausen , D. Weber , and R. E. Dunin‐Borkowski , “Wigner Distribution Deconvolution Adaptation for Live Ptychography Reconstruction,” Microscopy and Microanalysis 29, no. 3 (2023): 994–1008. 37749665 10.1093/micmic/ozad021

