# Supplementary material for: Gap‐Free Information Transfer in 4D‐STEM via Fusion of Complementary Scattering Channels
Source: Adv Sci (Weinh). 2026 Jul 23;13(56):e76620. doi: 10.1002/advs.76620 (PMC13393279; doi:10.1002/advs.76620)
Supplement: Supplementary file 1 [file ADVS-13-e76620-s001.pdf]

Supporting Information

Gap-free Information Transfer in 4D-STEM via Fusion of Complementary Scattering Channels

Shengbo You, Georgios Varnavides, Sagar Khavnekar, Nikita Palatkin, Sihan Shao, Mingjian Wu, Daniel Stroppa, Darya Chernikova, Baixu Zhu, Ricardo Egoavil, Stefano Vespucci, Xingchen Ye, Florian K. M. Schur, Erdmann Spiecker, Philipp Pelz*

Corresponding Author: Philipp Pelz (philipp.pelz@fau.de)

S1 Supplementary Figures

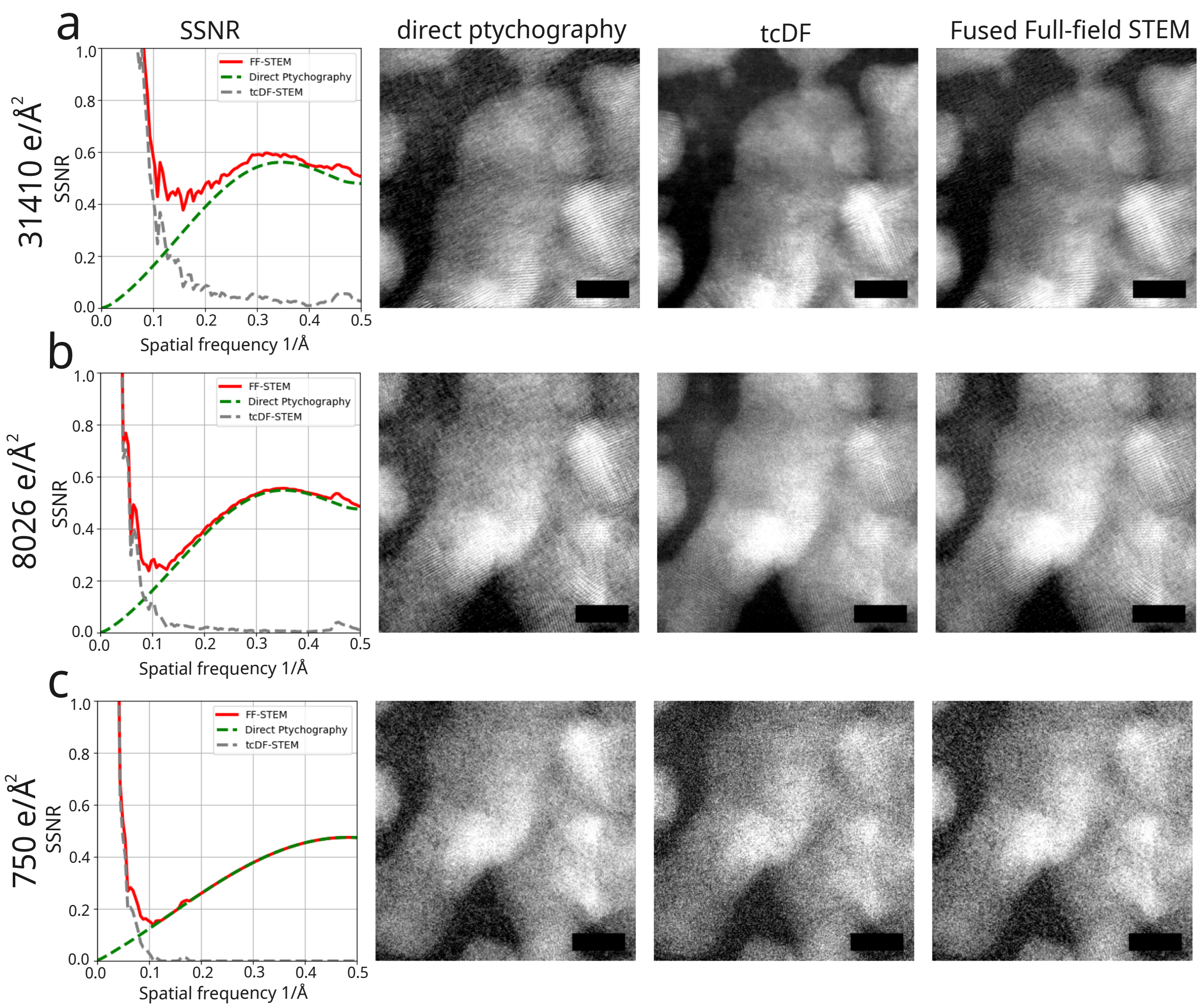

Figure S1. Effect of dose on contrast transfer in a single 4D-STEM experiment Dose-series acquired on a diffraction grating replica at 200 kV accelerating voltage and 15 mrad convergence semi-angle. All datasets were recorded with identical scan geometry (same scan step and number of scan positions), while the total electron dose was varied by adjusting beam current and dwell time: (a) 31410 e/Å^2, (b) 8026 e/Å^2, and (c) 4013 e/Å^2. Left column: spectral signal-to-noise ratio (SSNR) of direct ptychography (green), tilt-corrected dark-field (tcDF, gray), and fused FF-STEM (red). Right columns: corresponding real-space reconstructions from direct ptychography, tcDF, and FF-STEM. The direct ptychography SSNR shows only minor dependence on dose, with a slight reduction at high spatial frequencies at the lowest dose, consistent with increased shot noise. In contrast, the tcDF SSNR is strongly dose dependent: while it provides robust low-frequency transfer at high dose, its SSNR rapidly decreases at intermediate and high spatial frequencies as the dose is reduced. As a result, the FF-STEM reconstruction adapts accordingly via SSNR-based weighting, incorporating low-frequency tcDF information at high dose and progressively relying on direct ptychography as tcDF becomes noise-limited at low dose. The scale bars is 2nm.

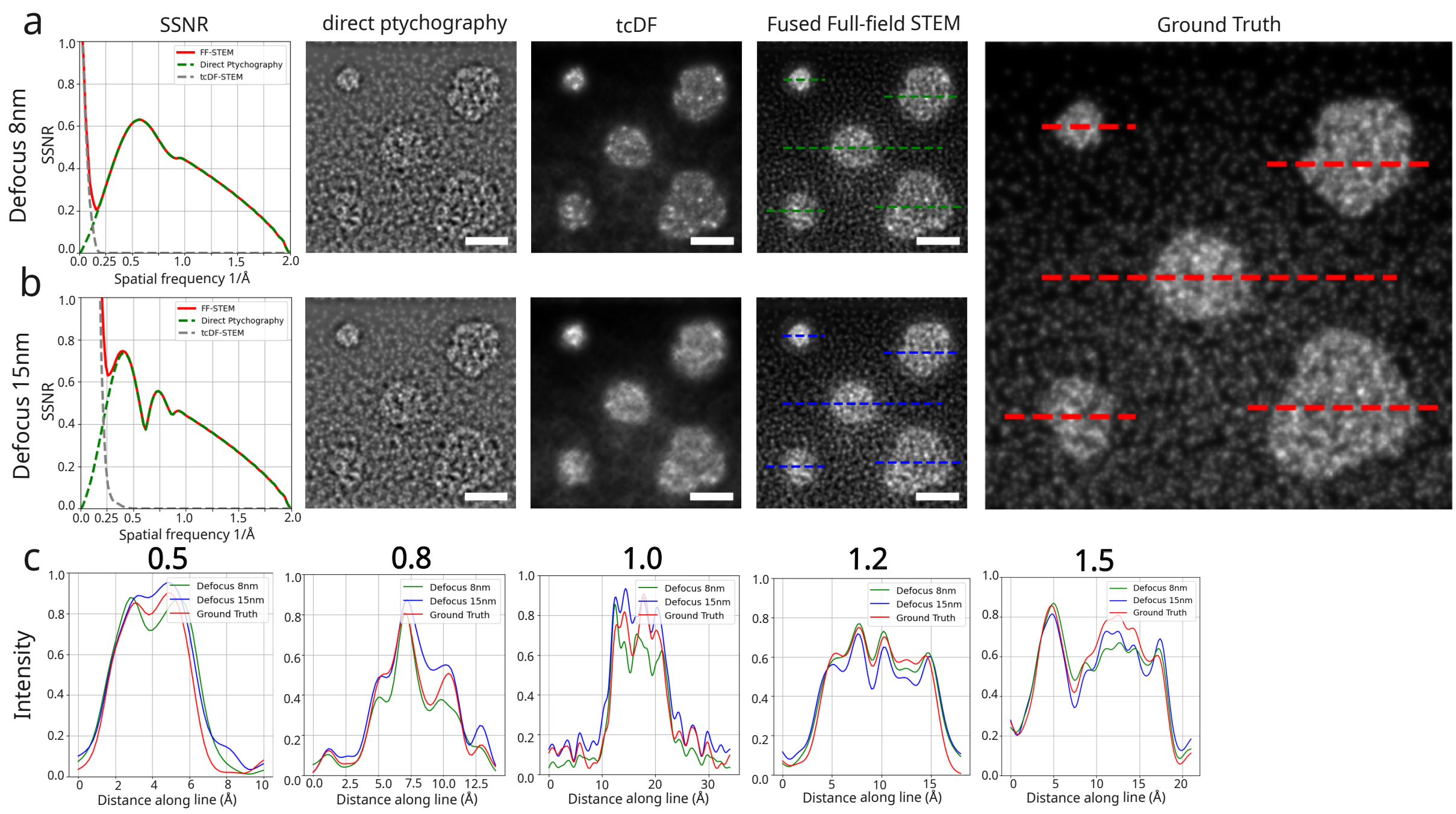

Figure S2. Systematic evaluation of FF-STEM transfer around 0.1 Å^{-1}using simulated mesoscale features. A simulated amorphous-carbon test volume was constructed to probe image transfer at spatial frequencies near 0.1 Å^{-1}, corresponding to real-space feature sizes on the order of 1 nm. The background consists of amorphous carbon with a thickness gradient increasing from 0 nm at the top to 3 nm at the bottom. Superimposed on this background are five irregular amorphous-carbon islands with characteristic diameters of 0.5 (top left), 0.8 (bottom left), 1.0 (middle), 1.2 (top right), and 1.5 (bottom right) nm, chosen to directly test the recovery of features in the spatial-frequency range relevant to the transfer dip at 0.1 Å^{-1} at Figure 1 in main text. Two 4D-STEM datasets were simulated from the same volume using probe defocus values of 8 nm and 15 nm. (a,b) For each defocus condition, the left panel shows the SSNR curves of direct ptychography, tcDF, and FF-STEM, followed by the corresponding direct ptychography, tcDF, FF-STEM, and ground-truth images. At 8 nm defocus, the FF-STEM SSNR exhibits a dip near 0.1 Å^{-1} whereas at 15 nm defocus this dip is reduced, demonstrating that the transfer minimum can be shifted or mitigated by changing the probe defocus. Despite these differences in spectral transfer, the fused reconstructions recover the shape and contrast of the mesoscale islands well in both cases. (c) Line profiles extracted along the marked dashed lines for each island size, comparing the FF-STEM reconstructions at 8 nm and 15 nm defocus with the ground truth. Across all island sizes, both defocus conditions reproduce the main real-space contrast variations closely, indicating that features around 1 nm are not strongly suppressed or smoothed out in the fused reconstruction, even when the transfer curve contains a local dip near 0.1 Å^{-1}. The scale bars in the images correspond to 1 nm.

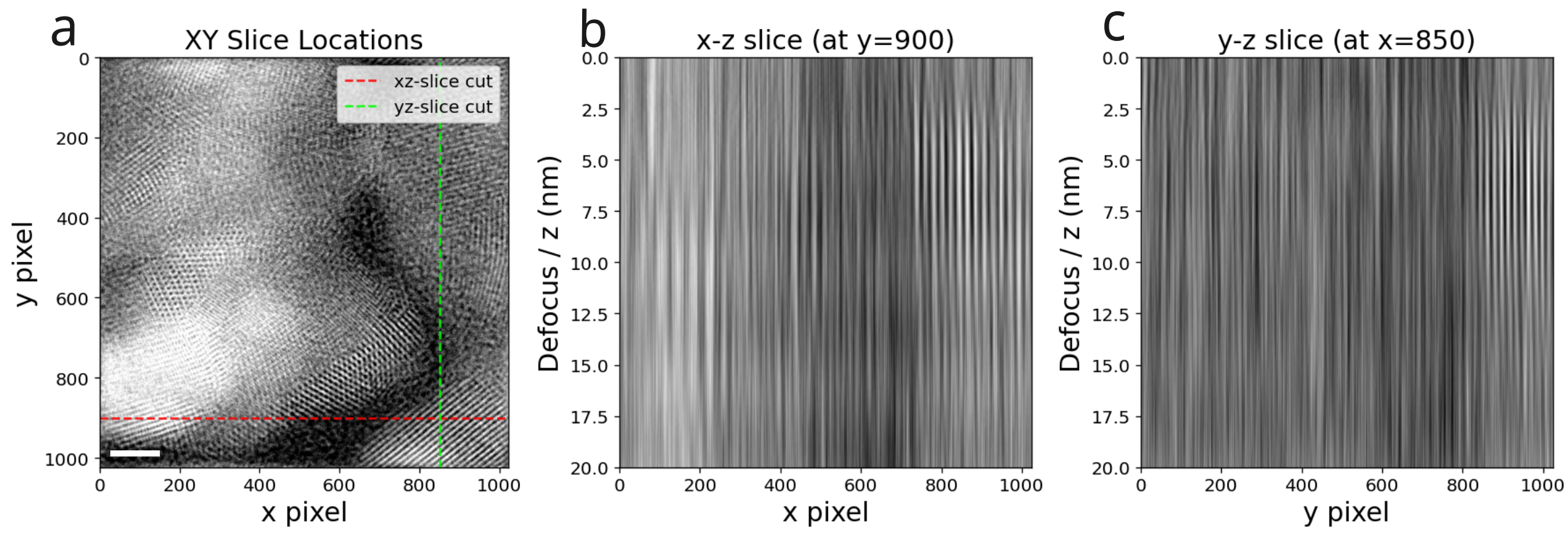

Figure S3. Quantitative assessment of depth-sectioning through a reconstructed focal stack. (a) FF-STEM reconstruction of diffraction grating data (Figure 4a) indicating the spatial locations used for axial cross-sections. The horizontal red dashed line marks the position for the x-z slice ($y = 900$), and the vertical green dashed line marks the position for the y-z slice ($x = 850$). (b) X-z cross-sectional slice through the focal stack, showing the signal evolution along the optical axis (defocus z). (c) Y-z cross-sectional slice corresponding to the $x = 850$ position. A Full Width at 80% Maximum (FW80M) analysis is performed and show the axial resolution being 4.8 nm. The scale bar in (a) corresponds to 2nm.

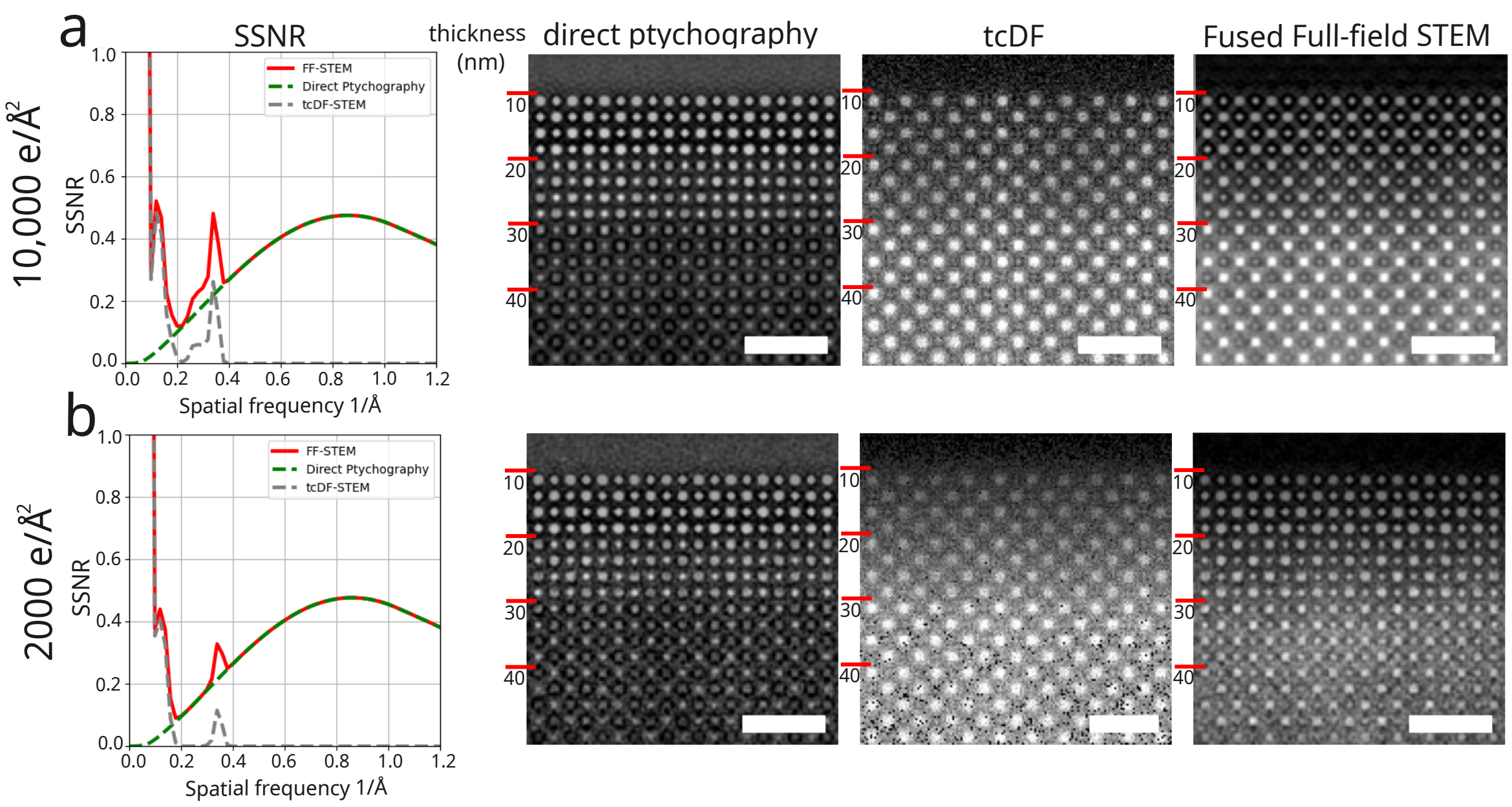

Figure S4. Thickness-dependent performance of FF-STEM on a simulated $SrTiO_3$ crystalline wedge Simulated $SrTiO_3$ wedge sample with thickness increasing from 10 to 40 nm, as indicated on the left. Each step corresponds to two unit-cell thickness increment along the wedge direction. For each dose condition, the left panel shows the SSNR curves of direct ptychography, tcDF, and FF-STEM, followed by the specimen thickness indicator, and the corresponding real space reconstructions. (a) simulation at 10,000 e/Å^2. (b) simulation at 2000 e/Å^2. Direct ptychography preserves atomic-column contrast at small and intermediate thicknesses but progressively loses quantitative interpretability at larger thicknesses due to the breakdown of the weak-phase approximation under strong dynamical diffraction. In contrast, tcDF retains visually strong heavy-atom column contrast even at the largest thicknesses. This apparent robustness arises because the dark-field signal is dominated by high-angle, largely incoherent scattering from strongly scattering atomic columns, which continues to produce intensity contrast even in the presence of multiple scattering. As a result, heavy columns remain visible ("look-nice") at large thickness. However, this contrast is not equivalent to a linear projection of the electrostatic potential: at large thickness, the tcDF intensity is strongly influenced by dynamical diffraction, channeling, thickness-dependent scattering, and detector-angle selection, and therefore does not provide quantitative atomic-level information. FF-STEM combines the complementary behavior of both channels, incorporating low-frequency contrast from tcDF and high-frequency information from direct ptychography where both remain reliable, while the thickest regions mark the expected breakdown regime of the linear fusion model. The scale bars is 1 nm.

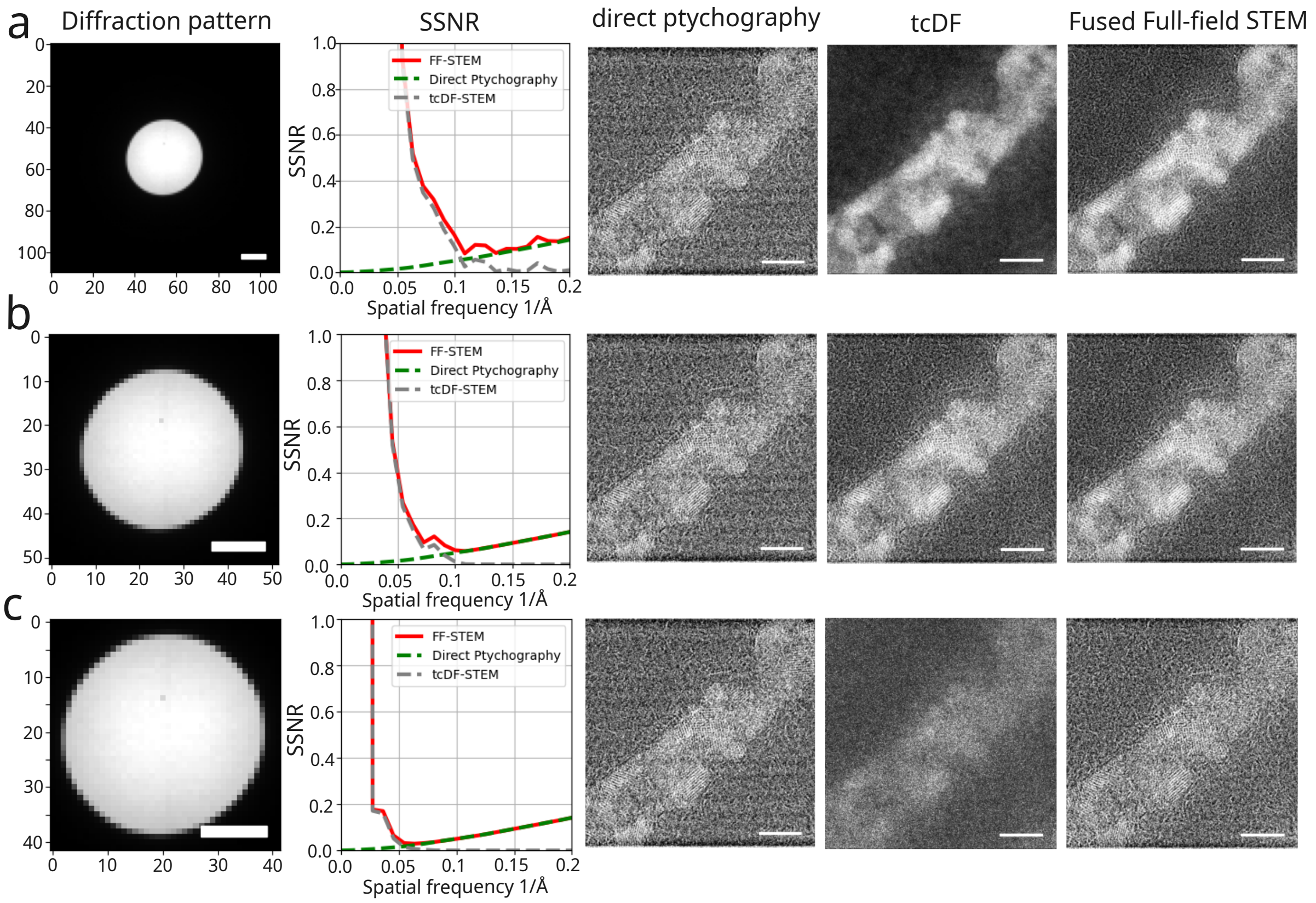

Figure S5. Influence of the maximum collected scattering angle on tcDF and FF-STEM reconstruction. Rows (a–c) show reconstructions obtained from the same 4D-STEM dataset after progressively restricting the detector to smaller angular ranges, as illustrated by the cropped mean diffraction patterns in the leftmost column. The second column shows the corresponding SSNR, followed by the reconstructed images from direct ptychography, tcDF, and FF-STEM. When a larger dark-field angular range is retained (a), the tcDF channel provides substantial low-spatial-frequency contrast, which is transferred to the fused reconstruction. As the maximum available scattering angle is reduced by cropping the dark-field region (b,c), the tcDF reconstruction progressively loses useful contrast and becomes less informative, which is reflected by the suppression of its SSNR and by the reduced low-frequency enhancement in FF-STEM. These results show that the benefit of FF-STEM depends on the availability of sufficiently high-angle scattered electrons: limiting the detector angular range weakens the complementary dark-field contribution and reduces the degree to which the low-frequency contrast gap can be filled. The scale bar in the diffraction patterns corresponds to 10 mrad, and the scale bars in the reconstructed images correspond to 5 nm.

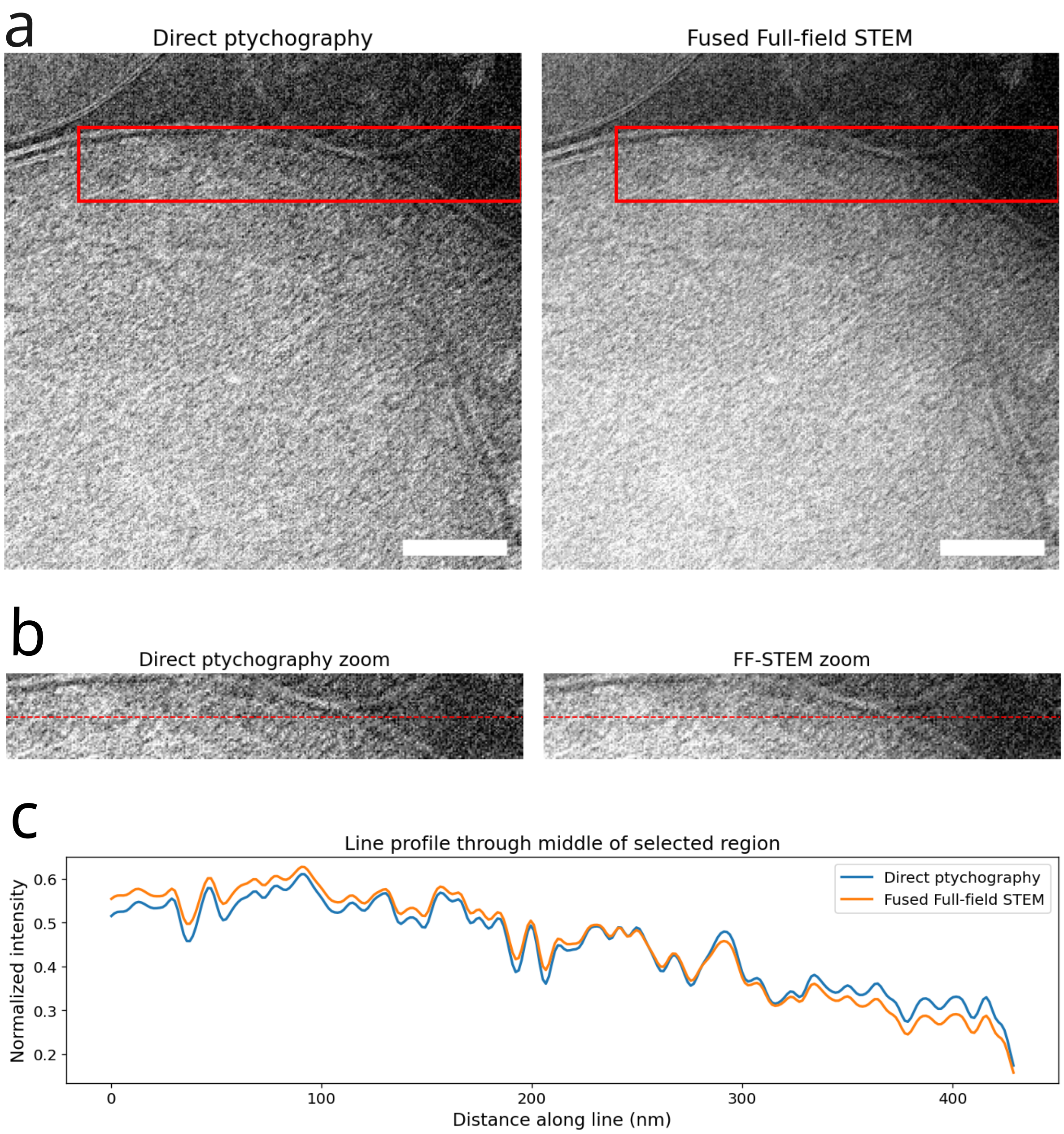

Figure S6. Direct side-by-side comparison of direct ptychography and FF-STEM for the mitochondrion dataset. (a) Direct ptychography and FF-STEM reconstructions of the same mitochondrion region displayed with identical field of view and contrast normalization. The red rectangles mark the region selected for direct comparison, containing both the specimen and surrounding background. (b) Enlarged views of the selected regions show that FF-STEM provides stronger separation between the mitochondrion and the background while preserving the same membrane-like structural features visible in direct ptychography. The red dashed line indicates the position used for the line-profile analysis. (c) Line profiles extracted from the same position in the two reconstructions. Compared with direct ptychography, FF-STEM shows higher intensity in the specimen-containing region and lower intensity in the background region, quantitatively supporting the improved sample–background contrast obtained by adding the low-frequency dark-field contribution. Scale bars: 100 nm.

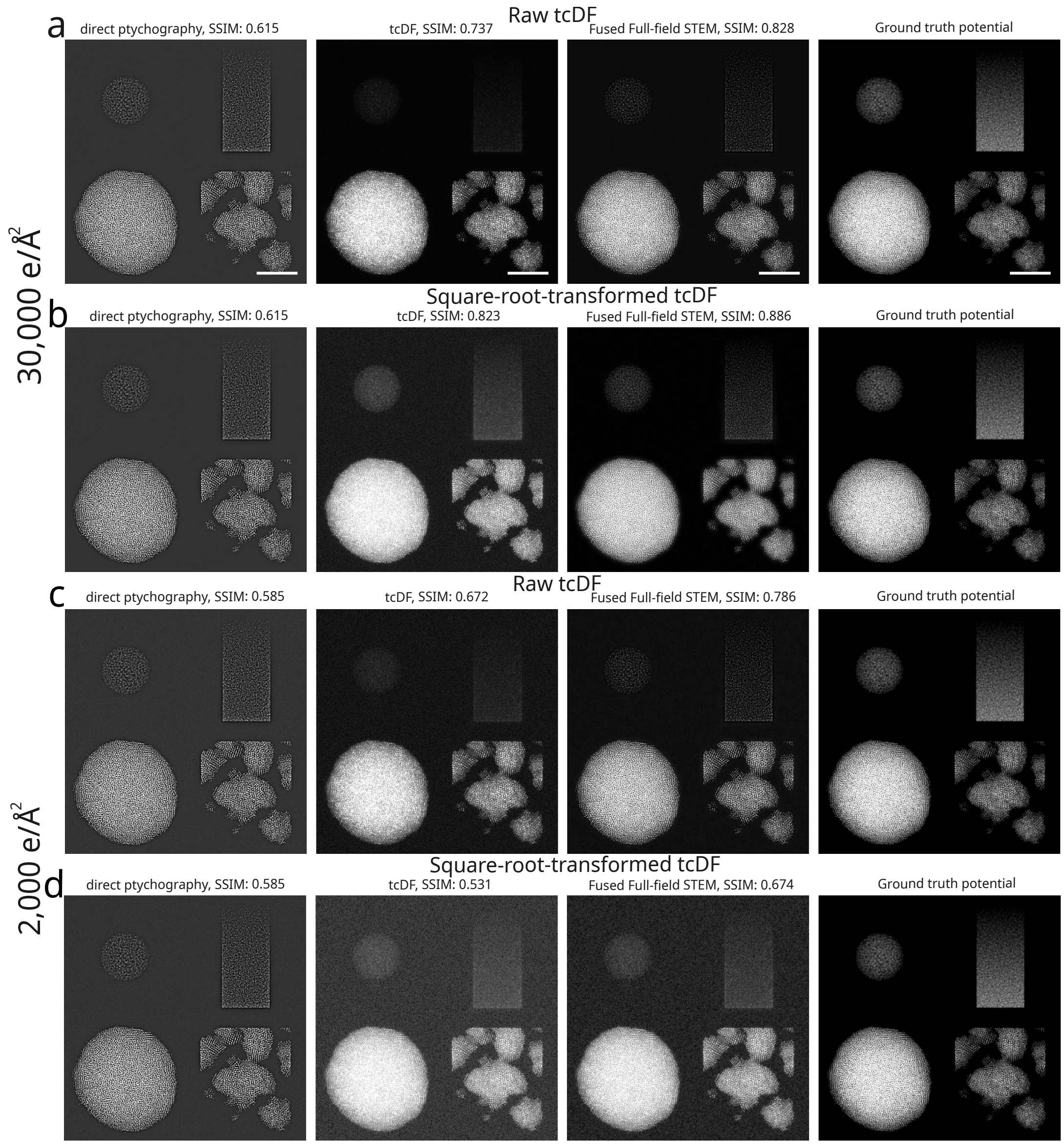

Figure S7. Effect of square-root transforming the tcDF channel in a multi-material 4D-STEM simulation. The simulated field of view contains four chemically distinct regions arranged in quadrants: a Si/O-containing model in the upper-left quadrant, an amorphous-carbon region in the upper-right quadrant, a high-Z Pd nanoparticle in the lower-left quadrant, and a Ta film/particle region in the lower-right quadrant. This mixed-Z specimen was designed to test whether the raw tcDF signal is dominated by strongly scattering high-Z regions, and whether applying a square-root transform to tcDF improves the balance of contrast between light- and heavy-element regions. Rows (a,b) show reconstructions at high dose, 30,000 e/Å^2. Rows (c,d) show the corresponding comparison at lower dose, 2,000 e/Å^2. For each dose, the first row uses the raw tcDF reconstruction for fusion, while the second row uses a square-root-transformed tcDF reconstruction. At high dose, the square-root transform compresses the strong high-Z tcDF contrast and enhances the visibility of weaker low-Z regions, leading to improved SSIM for both tcDF and FF-STEM. This indicates that nonlinear compression of the tcDF dynamic range can partially reduce heavy-element dominance when the dark-field signal has sufficiently high SNR. At lower dose, however, the square-root transform also amplifies weak-signal background fluctuations, increasing the diffuse background in the tcDF image and reducing the quality of the fused FF-STEM reconstruction. These results show that square-root-transformed tcDF can improve contrast balance in high-SNR conditions, but is not generally advantageous at low dose, where raw tcDF provides a cleaner and more robust input for SSNR-based fusion. Scale bars: 5 nm.
